# People are skeptical of headlines labeled as AI-generated, even if true or human-made, because they assume full AI automation

**DOI:** 10.1093/pnasnexus/pgae403

**Published:** 2024-10-01

**Authors:** Sacha Altay, Fabrizio Gilardi

**Affiliations:** Department of Political Science, University of Zurich, 8050 Zürich, Switzerland; Department of Political Science, University of Zurich, 8050 Zürich, Switzerland

**Keywords:** label, artificial intelligence, news, accuracy, social media

## Abstract

The rise of generative AI tools has sparked debates about the labeling of AI-generated content. Yet, the impact of such labels remains uncertain. In two preregistered online experiments among US and UK participants (*N* = 4,976), we show that while participants did not equate “AI-generated” with “False,” labeling headlines as AI-generated lowered their perceived accuracy and participants’ willingness to share them, regardless of whether the headlines were true or false, and created by humans or AI. The impact of labeling headlines as AI-generated was three times smaller than labeling them as false. This AI aversion is due to expectations that headlines labeled as AI-generated have been entirely written by AI with no human supervision. These findings suggest that the labeling of AI-generated content should be approached cautiously to avoid unintended negative effects on harmless or even beneficial AI-generated content and that effective deployment of labels requires transparency regarding their meaning.

**Significance Statement**

AI-generated content is proliferating online, and social media companies have started to label such content. In two experiments, we show that people are less likely to believe and share headlines labeled as “AI-generated,” even when the headlines are actually true or human-generated. This is due to unrealistic expectations that headlines labeled as AI-generated have been entirely written by AI with no human supervision. This suggests that the effective deployment of labels will require transparency regarding their meaning and should be approached cautiously to avoid unintended negative effects. Overall, the impact of the “AI-generated” labels is small, three times smaller than “False” labels. To maximize impact, false AI-generated content should be labeled as false rather than solely as AI-generated.

## Introduction

The rapid advancement and widespread availability of generative AI technologies have spurred debates among policymakers and digital platforms about the necessity of content moderation policies for AI-generated content, including the use of labels similar to those for fact-checked false content. While there is a growing consensus on the potential need for such labels, research examining their impact on user perception and content engagement remains scarce. Empirical investigation into the effects of these labels is important given that they may have unintended negative consequences, such as reducing belief in or the sharing of accurate content. Study 1 investigates how labeling headlines as AI-generated influences both the perceived accuracy of the headlines and participants’ intention to share them, while Study 2 explores the mechanisms responsible for these effects.

Labeling content at scale is challenging. If social media companies were to label AI-generated content, they would face at least two challenges. First, as with any kind of labeling, social media companies would not be able to label all AI-generated content and would inevitably miss some. This scenario has been studied in the case of misinformation and fact-checking. Past work on veracity labels has shown that labeling some false news as false, but not all of them, increases the perceived veracity of unlabeled false news and participants’ willingness to share them (1)—an effect dubbed the *implied truth effect*. Second, given the difficulty of detecting AI-generated content, some human-generated content would inevitably be mislabeled as AI-generated. Past work on veracity labels has shown that true news articles mislabeled as inaccurate were perceived as less credible (2), which is in line with a broader literature on the *tainted truth effect*, showing that inaccurate warnings about misinformation reduce belief in accurate information (3).

More broadly, being exposed to AI labels may act as a kind of warning that the online environment is saturated with AI-generated content and is not a reliable space for acquiring trustworthy information. While this may be beneficial within low-quality information ecosystems where information should be met with skepticism, it also poses the risk of fostering undue skepticism within high-quality information environments (4). For instance, research on misinformation has shown that common interventions against misinformation and tips to help people spot misinformation can reduce trust in legitimate news and the perceived accuracy of true news (5–8). Similarly, informing people about deepfake videos makes them more likely to believe that any video, even real ones, is fake (9). This consideration is particularly relevant given that, beyond concerns about misinformation (10), many worry that generative AI may erode trust in all digital content, even when accurate and reliable (11). It is thus important to ensure that the use of such labels will not sow distrust in all content.

Past work on “automated journalism” and “robot journalism” has shown that people rate human-written news as much more readable, of slightly higher quality, and as equally credible than computer-written news (for a meta-analysis, see 12). Experimentally manipulating whether an article is said to have been written by a human or a computer has a small but consistent effect, such that participants rate human-written articles more favorably (13). Similarly, van der Kaa and Krahmer (14) tested the effect of labeling computer-generated news articles as either computer-generated or human-generated. They found that while Dutch journalists rated articles labeled as human-generated more favorably, other Dutch participants tended to do the opposite, and rated articles labeled as computer-generated more favorably. More recent work has shown that, in the United States, human-generated headlines labeled as AI-generated are rated as less accurate than the same human-generated labeled as human-generated (an effect robust across true and false headlines; 15). Another study conducted in the United States found that full news articles labeled as AI-generated are perceived as less trustworthy (but not as less accurate or more unfair)—although this effect is greatly diminished when the list of sources used by the AI to generate the article is disclosed (16).

The present work builds on and extends existing work. First, we investigated the effect of labels on both perceived accuracy and sharing intentions—while Longoni et al. (15) and Toff and Simon (16) did not measure sharing intentions. Second, we tested the effect of the AI labels on both AI-generated headlines and human-generated headlines (and on true and false news)—while past studies focused exclusively on one type of content, such as human-generated headlines (15), or did not vary the veracity of the news articles (16). Third, we compared the effect of AI labels to the effect of false labels. Fourth, we investigated the potential unintended consequences of these labels on unlabeled content, trust in the news, and attitudes toward AI. Finally, we experimentally investigated the mechanisms responsible for the effect of the labels.

In Study 1, participants were randomly assigned to one of the five following conditions: (i) the Control Condition in which no headline was labeled, (ii) the correct label condition in which all AI-generated headlines were labeled, (iii) the missing label condition in which only half of AI-generated headlines were labeled, (iv) the noisy label condition in which half of AI-generated headlines were labeled and half of human-generated headlines were mislabeled, and (v) the false label condition in which false headlines were labeled as false. Participants were exposed to 16 headlines and rated either the perceived accuracy of the headlines or their intention to share them. Our main hypothesis, which we have broken down into three variants (H_1–3_), was that headlines labeled as AI-generated would be rated as less accurate and receives lower sharing intentions. We also hypothesized that exposure to headlines labeled as AI-generated would reduce trust in the news and journalists (H_4_)^a^. We found that labeling headlines as AI-generated reduced the perceived accuracy of the headlines and participants’ intention to share them, regardless of the headlines’ veracity (true *vs.* false) or origin (human- *vs.* AI-generated). The effect of labeling headlines as AI-generated (2.66pp) was three times smaller than the effect of labeling headlines as false (9.33pp).

In Study 2, we replicated the main effect of Study 1 and investigated why people are skeptical of headlines labeled as AI-generated. To do so, we introduced three new conditions in which participants were provided with definitions explaining what it meant for a headline to be AI-generated. In the Weak Condition, participants were told that AI was used to improve the clarity of the text and adapt its style. In the Medium Condition, participants were told that AI contributed more substantially by writing a first draft of the article, while in the Strong Condition AI chose the topic of the article and wrote the whole article. We found that people believe less headlines labeled as AI-generated because they assume that headlines labeled as such have been fully generated by AI, such as AI selecting the topic of the article and writing it. Indeed, the labels only induced skepticism when participants were given no AI definitions (just like in Study 1) or when they were given a Strong AI definition.

These findings imply that care is needed when labeling AI-generated content to prevent adverse effects on nonharmful or even useful AI material. They suggest that that if AI-generated content is harmful, it should be explicitly labeled as such, rather than simply being tagged as AI-generated. Moreover, they highlight the importance of transparency regarding the meaning of the labels, to avoid users making wrong assumptions and react in unintended ways to them.

## Study 1

### Methods

#### Participants

Between 2023 August 31 and 2023 September 4, we recruited 1,976 participants in the United States via Prolific and excluded one participant who failed the attention check and two participants who took the survey more than once (992 women and 987 men; 997 Independents, 498 Republicans, 484 Democrats; *M*_age_ = 40.30, SD_age_ = 13.55). The sample was balanced in terms of gender and political orientation to match national distributions. Participants were paid £0.90 (£9/h) to complete the study (for a median completion time of 6 min). Focusing on the United States in Study 1 allows us to benchmark our results to past work conducted in the United States with comparable methods (15–17).

#### Hypotheses

In line with past work on automated journalism and AI aversion (18), we hypothesized that headlines labeled as AI-generated would be rated as less accurate and receive lower sharing intentions. We tested the effect of labeling headlines as AI-generated in three ways: first, by comparing AI-generated headlines labeled as AI to unlabeled AI-generated headlines, when all AI-generated headlines are labeled as such; second, by comparing AI-generated headlines labeled as AI to unlabeled AI-generated headlines, when some AI-generated headlines are not labeled and when some human-generated headlines are mislabeled; and third, by comparing human-generated headlines labeled as AI to unlabeled human-generated headlines. The distinction between H_1_ and H_2_ is subtle: H_1_ mimics a “perfect” social media environment in which all AI-generated headlines are labeled as such and none of the human-generated headlines are mislabeled, whereas H_2_ reflects a more ecologically valid environment in which some AI-generated headlines are not labeled and some human-generated headlines are mislabeled. H_3_ investigates one potential unintended consequence of labeling if deployed at scale: some human-generated content may be mislabeled as AI-generated. In line with the *tainted truth effect* (2, 3), we predicted that human-generated headlines mislabeled as AI-generated would suffer from a decrease in perceived accuracy and sharing intentions.

H_1_: AI-generated headlines labeled as AI-generated will be rated as less accurate and be less shared than unlabeled AI-generated headlines (when all AI-generated headlines are labeled as such).

H_2_: AI-generated headlines labeled as AI-generated will be rated as less accurate and be less shared than unlabeled AI-generated headlines (when not all AI-generated headlines are labeled and when some are mislabeled).

H_3_: Human-generated headlines labeled as AI-generated will be rated as less accurate and be less shared than human-generated headlines not labeled.

Given the negative perception of AI-generated headlines, and past work on warning about misinformation and deepfakes (5–9), we investigated the potential unintended consequences that AI-generated labels may have on the broader information ecosystem. We hypothesized that being exposed to headlines labeled as AI-generated would reduce trust in the news and journalists (H_4_).

H_4_: Participants exposed to headlines labeled as AI-generated will report lower trust in the news and journalists compared to participants in the Control Condition not exposed to any label.

We investigated the effect of the labels on attitudes toward AI and on support for labeling (RQ_1_). We did not have preregistered expectations about these effects or their directionality. For instance, exposure to headlines labeled as AI-generated could make people more worried about AI-generated news or increase support for the labeling of such news.

RQ_1_: What is the effect of being exposed to headlines labeled as AI-generated on attitudes toward AI, the news, and support for labeling?

To benchmark the effect of AI-generated labels, we compared it to the well-documented effect of false labels (i.e. labeling content as “False”). This comparison complements past work based on self-report measures (19) and allows us to causally test whether participants equate “AI-generated” with “False” and whether they are similarly suspicious of content labeled as “AI-generated” or “False.”

RQ_2_: Is the effect of AI-generated labels on accuracy ratings and sharing intentions similar to the effect of false labels?

Finally, following the literature on the *implied truth effect* (1), we report a nonpreregistered research question on the effect of AI-generated labels on unlabeled AI-generated headlines (RQ_2_).

RQ_3_: Will the presence of AI-generated headlines labeled as AI-generated increase the accuracy and sharing ratings of unlabeled AI-generated headlines?

#### Design and procedure

Participants were randomly assigned to one of the five conditions outlined below. All participants saw 16 headlines presented in a Facebook format (a headline with an image) without a source to allow for the use of AI-generated headlines. Half of participants rated the accuracy of the headlines (“How accurate is the claim in the above headline?” from “Certainly False” [1] to “Certainly True” [6]—a commonly used question in the literature on news judgments; 20), while the other half reported how willing they would be to share the headlines (“If you were to see this post online, how likely would you be to share it?” from “Extremely unlikely” [1] to “Extremely likely” [6]—a commonly used question in the literature on labels and interventions against misinformation; 21).

We created a pool of 16 news headlines, half true and half false. True headlines were found on mainstream US News outlets while false headlines were found on fact-checking websites such as PolitiFact and Snopes. The AI-generated headlines were created by asking chatGPT (version 3.5) to create headlines based on the full text of the news articles, the specific prompt was: “Can you create a headline and a 1-sentence led based on this news article: [*copy paste the full text of the article*].” All participants saw the same 16 stories, but we manipulated between-participants which version of the headlines they saw (human- or AI-generated) by creating sets of headlines with different versions of the stories. For example, all participants read a story about the Hollywood strikes (among 15 other stories), but some participants saw the human-generated version of the story while others saw the AI-generated version. No participant saw twice the same story.

Recently, Epstein et al. (19) surveyed participants from the United States, Mexico, and Brazil about their perceptions of labels applied to AI-generated content. They found that the label “AI-generated” is considered to be the most appropriate label; we used this label in the present experiment. In Study 1, we do *not* explain to participants what “AI-generated” means given that social media platforms have and will implement these labels without explaining what they mean precisely. For instance, in 2023 September, TikTok has been using the automatic label “AI-generated” (and the self-disclosed label “Creator labeled as AI-generated). Similarly, in 2024 Facebook and Instagram plan on using very generic labels such as “AI info.” Because users may interpret labels in different ways, it is important to understand the effect of generic labels, which reflects how such interventions are experienced by users in real-world settings.

In all conditions, one-fourth of the headlines were true and human-generated, one-fourth were true and AI-generated, one-fourth were false and human-generated, and one-fourth were false and AI-generated. The key experimental manipulation across conditions is the label applied to the headlines (see Table 1).

- *Control condition*: no headlines are labeled.
- *Correct labels condition*: all AI-generated headlines are labeled as “Generated by Artificial Intelligence.”
- *Missing labels condition:* only four out of eight AI-generated headlines are labeled as “Generated by Artificial Intelligence.”
- *Noisy labels condition:* four out of eight human-generated headlines are labeled as “Generated by Artificial Intelligence” and only four out of eight AI-generated headlines are labeled as “Generated by Artificial Intelligence.”
- *False labels condition*: all false headlines are labeled as false, and no AI-generated labels are applied.

Before rating the headlines, participants reported the extent to which they trust (i) most news most of the time, (ii) journalists most of the time, and (iii) news on social media most of the time (from “Strongly disagree” [1] to “Strongly agree” [7], with “Neither agree nor disagree” as the middle point [4]; 22). They then completed an attention check requiring them to read instructions hidden in a paragraph and write “I pay attention.”

**Table 1.** Overview of labeling of the headlines across conditions.

	AI-generated headlines		Human-generated headlines	
	True headlines	False headlines	True headlines	False headlines
Control	No label	No label	No label	No label
Correct labels	All labeled as AI	All labeled as AI	No label	No label
Missing labels	Half labeled as AI	Half labeled as AI	No label	No label
Noisy labels	Half labeled as AI	Half labeled as AI	Half labeled as AI	Half labeled as AI
False labels	No label	All labeled as false	No label	All labeled as false

Each row corresponds to a distinct control or treatment condition.

**Fig. 1.**
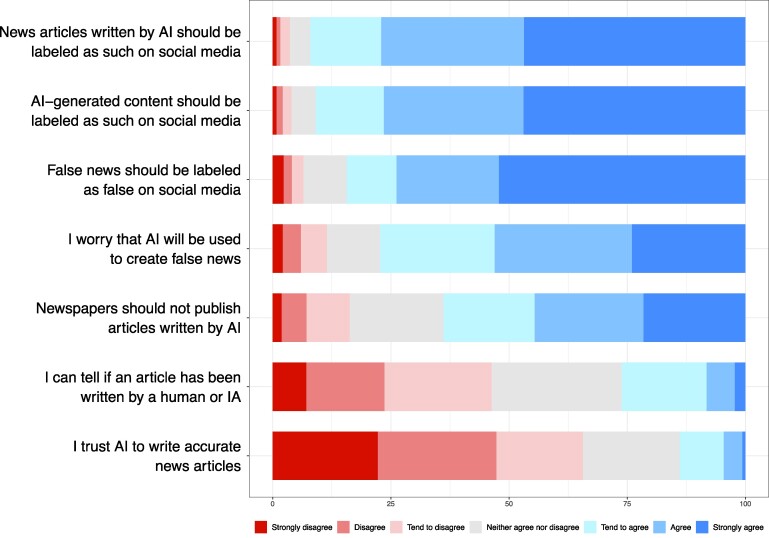
Attitudes toward AI, news, and content labeling on social media.

After rating the headlines, participants reported their trust in news, journalists, and news on social media again. Next, participants reported the extent to which they agreed with four statements about artificial intelligence and news, and the extent to which they are in favor of labeling various kinds of content (see Fig. 1).

Information about political orientation (identifying as an Independent, Democrat, or Republican), gender (men or women), and age was retrieved via Prolific. At the end of the survey, participants were debriefed about the purpose of the study and warned about their exposure to false news. The study received ethical approval from the University of Zürich PhF Ethics Committee (ethics approval no. 23.04.17). All participants gave their informed consent.

#### Statistical analyses (Studies 1 and 2)

We use an alpha of 5% as the threshold for statistical significance. Values in square brackets are 95% CIs. To test the effect of labeling on accuracy and sharing ratings, we analyzed the data at the response level (*N*_observations_ = 31′600 in Study 1 and *N*_observations_ = 60′020 in Study 2) and conducted linear mixed-effects models with participants, headlines, and news set as random effects. The effect on attitudes was analyzed at the participant level and tested with OLS linear regressions. In all the models, age, gender, and political orientation were added as predictors, together with the veracity of the headline (true or false), the type of dependent variable (sharing or accuracy), and Condition.

### Results

#### Descriptive information

Before diving into the results, we report descriptive information about the accuracy and sharing ratings of the headlines, and participants’ attitudes, measured post-treatment. In the control condition, participants rated the (original) human-generated headlines as less accurate than the AI-generated versions of the headlines (*b* = −0.23 [−0.31, −0.16]), but were not significantly more likely to share the human-generated headlines (*b* = −0.04 [−0.12, 0.04]). Regarding veracity, participants rated true headlines as much more accurate than false headlines (*b* = 1.32 [1.24, 1.42]) and were slightly more likely to share the false headlines (*b* = 0.09 [0.01, 0.17]).

In Fig. 1, we offer a descriptive overview of participants’ attitudes. We can see that participants were generally worried that AI will be used to create false news and did not trust AI to write accurate news articles. Most participants were not confident in their ability to tell if an article has been written by a human or AI. Finally, the large majority of participants were in favor of labeling false news as false, labeling AI-generated content as AI-generated, and labeling AI-generated news as AI-generated.

After the treatment, participants were asked about the effect they thought the AI-generated labels would have on their evaluations of the headlines. Overall, 59.8% of participants estimated that the labels would reduce their intention to share the headlines, and 62.3% that the labels would reduce the perceived veracity of the headlines (other participants mostly estimated null effects). Condition, and the type of dependent variable (accuracy or sharing), had no statistically significant effect on this outcome (see Supplementary material and Fig. S7 for more information).

#### What is the effect of labeling AI-generated headlines as AI-generated? (H_1_)

**Fig. 2.**
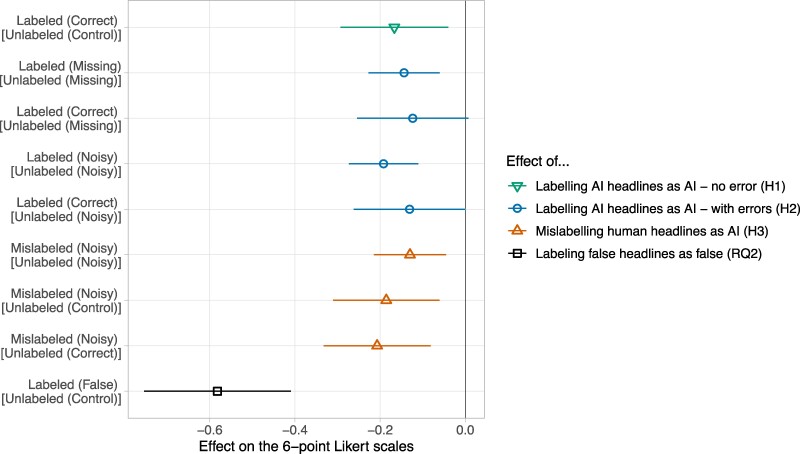
An overview of treatment effects. The first estimate (the inverted triangle) represents the effect of labeling AI headlines when all headlines are perfectly labeled (H_1_). The next four estimates (the circles) represent the effect of labeling AI headlines when not all headlines are perfectly labeled (H_2_). The next three estimates (the triangles) represent the effect of mislabeling human headlines (H_3_). The last estimate (the square) represents the effect of labeling false headlines as false (RQ_2_).

To test H_1_, we compared the sharing and accuracy ratings of nonlabeled AI-generated headlines in the Control condition to the ratings of labeled AI-generated headlines in the Correct labels condition (see Fig. 2). In line with H_1_, we found that labeling AI-generated headlines as AI-generated reduced sharing and accuracy ratings by.17 points [−0.29, −0.04], *P* = 0.010 on the 6-point scale.

The effect was mostly driven by a decrease in the perceived accuracy of the headlines (*b* = −0.21 [−0.33, −0.08], *P* = 0.002) rather than a decrease in sharing intentions (*b* = −0.12 [−0.34, 0.10], *P* = 0.27)—yet, this difference is not statistically significant (*b* = −0.09 [−0.16, 0.34], *P* = 0.49). The effect was significant, and of similar size, across true and false headlines (see Fig. S2).

#### What is the effect of labeling AI-generated headlines as AI-generated in more realistic environments? (H_2_)

While conceptually similar to H_1_, H_2_ investigates the effect of labeling AI-generated headlines when not all headlines are perfectly labeled. Instead of estimating the effect of labeling by taking as a reference unlabeled AI-generated headlines in the Control condition, H_2_ takes as a reference unlabeled AI-generated headlines in the Missing labels condition and in the Noisy labels condition. These comparisons allow us to estimate the effect of labeling in more realistic settings when not all AI-generated headlines are labeled (Missing labels condition) and when some headlines are mislabeled (Noisy labels condition). Overall, we found that the effect of labeling AI-generated headlines is similar in realistic (H_2_) and unrealistic settings (H_1_) (see Fig. 2).

First, we compared unlabeled AI-generated headlines in the Missing labels condition to (i) labeled AI-generated headlines in the Missing labels condition and (ii) labeled AI-generated headlines in the Correct label condition. We found that AI-generated headlines labeled as AI-generated in the Missing labels condition (*b* = −0.14 [−0.23, −0.06], *P* < 0.001), but not in the Correct labels condition (*b* = −0.12 [−0.25, 0.01], *P* = 0.064), received lower accuracy and sharing ratings than unlabeled AI-generated headlines in the Missing labels condition—although the size of the effects is similar and not statistically different from each other.

Second, we compared unlabeled AI-generated headlines in the Noisy labels condition to (i) labeled AI-generated headlines in the Noisy labels condition and (ii) labeled AI-generated headlines in the Correct label condition. We found that AI-generated headlines labeled as AI-generated in the Noisy labels condition (*b* = −0.20 [−0.28, −0.11], *P* < 0.001), but not in the Correct labels condition (*b* = −0.13 [−0.26, 0.001], *P* = 0.056), received lower accuracy and sharing ratings than unlabeled AI-generated headlines in the Noisy labels condition—although the effect goes in the same direction.

Overall, these effects were stronger on the perceived accuracy of the headlines than on sharing intentions (see Fig. S1), while they were similar across true and false headlines (see Fig. S2).

#### What is the effect of mislabeling human-generated headlines as AI-generated? (H_3_)

To test H_3_, we compared the ratings of human-generated headlines mislabeled as AI-generated in the Noisy labels condition to unlabeled human-generated headlines in (i) the Noisy labels condition, (ii) the Control condition, and (iii) the Correct labels condition. All three comparisons show that mislabeling human-generated headlines as AI-generated reduced sharing and accuracy ratings (see Fig. 2).

Labeling human-generated headlines as AI-generated reduced perceived accuracy and sharing intentions by.12 points [−0.20, −0.04] within the Noisy labels condition, by.18 points [−0.31, −0.06] when compared with the Control condition, and by.20 points [−0.33, −0.08] when compared with the Correct labels condition.

The size of these effects was similar across sharing intentions and accuracy ratings (see Fig. S1) and across true and false headlines (although the effects tended to be stronger among true news, see Fig. S2).

#### What is the effect of the labels on attitudes? (H_4_ and RQ_1_)

We hypothesized that exposure to news labeled as AI-generated would reduce trust in the news and journalists (H_4_). Our results do not support (H_4_), such that levels of trust post-treatment were similar in the Control condition and the treatments (ps > 0.60).

We formulated research questions regarding the effect of the labels on attitudes toward news and AI, and the level of support for labeling. Our results show no statistically significant differences across conditions (see Figs. S4–S6).

#### Comparing the effect of AI-generated labels to the effect of false labels (RQ_2_)

This last preregistered analysis aims at benchmarking the effect of AI-generated labels compared with the well-documented effect of false labels. First, we estimated the raw effect of labeling false headlines as false by comparing ratings of the false headlines (in the False labels condition) to ratings of false headlines in the Control condition. We found that the false labels reduced accuracy and sharing ratings by 0.56 points [−0.70, −0.42]. The false labels had a similar effect on the perceived accuracy of the headlines (*b* = 0.58 [−0.75, −0.41]) and sharing intentions (*b* = 0.52 [−0.73, −0.30]).

Second, we estimated the difference between the effect of false labels and AI-generated labels. To do so, we compared the ratings of false AI-generated headlines labeled as false (in the False labels condition) to the ratings of false AI-generated headlines labeled as AI-generated (in the Correct condition). We found that the false labels reduced sharing and accuracy ratings by.41 points [0.27, 0.56] more than the AI labels. This difference was similar across accuracy ratings (*b* = 0.43 [0.24, 0.62]) and sharing intentions (*b* = 0.38 [0.16, 0.60]).

#### Estimating spillover effects of the labels on unlabeled headlines—such as the implied truth effect (RQ_3-_exploratory)

In this section, we tested whether the presence of labels in the environment affected headlines that were not labeled. To do so, we compared ratings of unlabeled headlines across conditions. We found that the presence of AI-generated labels had no statistically significant effect on unlabeled headlines—regardless of whether the headlines were human- or AI-generated (see Fig. S3). Similarly, the presence of false labels had no statistically significant effect on unlabeled true headlines.

## Study 2

Study 2 replicates H_1_ and investigates the mechanisms behind the effect observed in Study 1: why are people skeptical of headlines labeled as AI-generated? We introduce three new conditions in which participants are given definitions of what it means for a headline to be AI-generated. In the Weak Condition, participants were told that AI was used to improve the clarity of the text and adapt its style. In the Medium Condition, participants were told that AI contributed more substantially by writing a first draft of the article, while in the Strong Condition participants were told that AI chose the topic of the article and wrote the whole article.

### Methods

#### Participants

Between the 2024 March 20 and the 2023 March 21, we recruited 3,003 participants in the United States and United Kingdom via Prolific and excluded two participants who failed the attention check (1,498 women and 1,499 men; *M*_age_ = 40.30, SD_age_ = 13.55). We recruited participants from the United Kingdom to test the generalizability of our findings outside of the United States. Past work on the effect of labels has exclusively focused on the unusual case of the United States (15–17), as has research on related topics such as digital media and democracy (23) or news judgments (20). Participants from the United Kingdom help us address some of the limitations of US-centric studies.

The sample was balanced in terms of gender and political orientation (one-third left, one-third right, and one-third center/independent). Participants were paid £0.90 (£9/h) to complete the study (for a median completion time of 6 min).

#### Hypotheses

Just like in Study 1, we tested the effect of the labels on accuracy ratings and sharing intentions (H_1_), the effect of the labels on trust in the news (H_4_), and potential spillover effects of the labels on unlabeled headlines (RQ_4_). In addition, Study 2 tests whether stronger definitions of AI-generated headlines, in which AI selected the topic of the article and wrote it, have stronger effects on sharing and accuracy ratings than weaker definitions of AI-generated headlines, in which AI played a smaller role by improving the clarity and style of the text, or wrote a first draft (H_2_).

H2: Stronger definitions of AI-generated headlines will have stronger effects on accuracy and sharing ratings than weaker definitions.

We also tested how participants implicitly and explicitly understand what it means for a headline to be AI-generated. To implicitly measure expectations about AI-generated headlines, we compared sharing and accuracy ratings of labeled AI headlines in the No Definition Condition to ratings of labeled AI headlines in the Weak, Medium, and Strong Conditions (RQ_1_). To explicitly measure expectations about AI-generated headlines, we asked participants (post-treatment) what they thought it meant for a headline to be AI-generated (RQ_2_).

RQ_1_: How do participants implicitly understand what it means for a headline to be AI-generated?

RQ_2_: How do participants explicitly understand what it means for a headline to be AI-generated?

Finally, we measured what AI-uses participants thought justified labeling a headline as AI-generated (RQ_3_). For instance, if AI was used to write the first draft of an article, should the headline be labeled as AI-generated?

RQ_3_: What kinds of AI uses deserve to be labeled?

#### Design and procedure

The design and procedure are similar to Study 1, except that participants saw 10 headlines instead of 16 (as 6 of them were outdated) and that participants were asked both how willing they would be to share the headlines (always first) and how accurate they found the claim in the headline. Moreover, we changed the wording of the labels from “Article generated by Artificial Intelligence” to “Text generated by Artificial Intelligence.”

Before rating the headlines, in all conditions, participants were told “Next, you will be presented with 10 news headlines and asked to answer a few questions about them. Some headlines may be human-generated while others may be generated by artificial intelligence (AI). If you see AI-generated headlines, they will be explicitly labeled as such.” Participants were randomly assigned to one of the following conditions:

- *Control Condition*: no label.
- *No Definition Condition*: all AI-generated headlines are labeled.
- *Weak Condition*: all AI-generated headlines are labeled, and participants are told that AI-generated means that: “(i) a journalist selected the topic of the article and wrote the article, and (ii) AI was used to improve the clarity of the text and adapt it to the style of the news outlet.”
- *Medium Condition*: all AI-generated headlines are labeled, and participants are told that AI-generated means that: “(i) a journalist selected the topic of the article, and (ii) AI wrote a first draft of the article based on sources provided by the journalist.”
- *Strong Condition*: all AI-generated headlines are labeled, and participants are told that AI-generated means that: “(i) AI selected the topic of the article, and (ii) AI wrote the whole article.”

Right after receiving these instructions, participants in the Weak, Medium, and Strong conditions were asked what AI-generated means based on the definition that they just read. Then, we repeated the definitions to make sure that participants paid attention to it.

After rating the 10 headlines, participants were asked what they thought AI-generated means and what kinds of AI-generated uses deserved to be labeled (see Figs. S11 and S12). Participants answered the following questions “In general, outside of this study, when a headline is labeled as “AI-generated,” do you think it means that:” and “In general, outside of this study, do you think that headlines should be labeled as AI-generated when:”. They indicated the extent to which they agree (from [1] “Strongly disagree’ to [7] “Strongly agree’, with [4] “Neither agree nor disagree’ as the middle option) with each of the following:

“AI summarized a news article to create the headline”

“AI shortened the article for social media”

“AI was used to fact-check the content of the article”

“AI wrote the whole article”

“AI wrote a first draft of the text”

“AI improved the clarity and style of the text”

“AI selected the topic of the article”

The question about the meaning of AI-generated was asked before the question about whether such uses should be labeled. In both cases, participants were told what labels mean: “(By labeling we mean adding a label or a tag next to the headline to indicate that the headline has been AI-generated).” We selected the response options based on current uses of AI systems by major news organizations in the United States, the United Kingdom, and Germany (24)—such as fact-checking, draft writing, reformatting or summarization. We also added some noncommon uses, such as AI writing the full article, as we suspected that people expect AI to be used this way. Such maximalists AI-uses with little to no human supervision may be common in some low-quality news outlets, but major news organizations that capture the vast majority of news traffic seem to use AI in much more nuanced and supervised ways (24).

### Results

#### What is the effect of labeling AI-generated headlines as AI-generated? (H_1_)

To test H_1_, we compared the sharing and accuracy ratings of unlabeled AI-generated headlines in the Control Condition to the ratings of labeled AI-generated headlines in the No Definition Condition. In line with H_1_, we found that labeling AI-generated headlines reduced the perceived accuracy of the headlines and participants’ willingness to share them by.11 points [−0.19, −0.03], *P* = 0.006 on the 6-point scales.

The effect sizes were similar across accuracy ratings (*b* = −0.12 [−0.20, −0.04], *P* = 0.002) and sharing intentions (*b* = −0.10 [−0.21, 0.02], *P* = 0.085), but just like in Study 1, only the decrease in perceived accuracy was statistically significant. When breaking down the results by country and sharing *vs.* accuracy, we see that all estimates are negative but only the accuracy estimate in the United Kingdom is statistically significant (see Fig. S8).

#### Stronger definitions of AI-generated headlines will have stronger effects on accuracy and sharing ratings than weaker definitions (H_2_)

To test H_2_, we compared the ratings of labeled AI-generated headlines in the Weak Condition to the ratings of labeled AI-generated headlines in the Strong Condition. In the Weak Condition, participants were told that AI improved the clarity and style of the text, while in the Strong Condition, participants were told that AI selected the topic of the article and wrote it.

In line with H_2_, we found that the labels reduced accuracy and sharing ratings more in the Strong Condition than in the Weak Condition (*b* = −0.12 [−0.20, −0.05], *P* = 0.002). The effect was mostly driven by a reduction in perceived accuracy (*b* = −0.17 [−0.25, −0.10], *P* < 0.001) rather than a reduction in sharing intentions (*b* = −0.08 [−0.18, −0.03], *P* = 0.18). When breaking down the results by country and sharing *vs.* accuracy, we see that all estimates are negative but only the accuracy estimates in the United Kingdom and United States are statistically significant (see Fig. S9).

We also compared the effect of the Medium Condition to the Weak and Strong Conditions. We found that the Medium Condition had a weaker effect on accuracy and sharing ratings than the Strong Condition (*b* = −0.09 [−0.17, −0.01], *P* = 0.025), while it did not statistically differ from the Weak Condition (*b* = 0.03 [−0.04, 0.11], *P* = 0.38).

#### The labels will reduce trust in the news (H_3_)

In line with Study 1, we found that being exposed to headlines labeled as AI-generated did not significantly affect participants’ trust in the news (see Fig. S13).

#### How do participants implicitly understand what it means for a headline to be AI-generated? (RQ_1_)

To test RQ_1_, we compared the ratings of headlines labeled as AI-generated when participants were given no definitions of AI-generated headlines (No definition Condition) to the ratings of headlines labeled as AI-generated when participants were given definitions of AI-generated headlines.

We found that the labels had weaker effects in the Weak Condition (*b* = −0.14 [−0.22, −0.07], *P* < 0.001) and in the Medium Condition (*b* = −0.11 [−0.19, −0.03], *P* = 0.006) compared with the No definition Condition. However, we found no statistical difference between the ratings in the Strong Condition and the ratings in the No definition Condition (*b* = −0.02 [−0.10, 0.06], *P* = 0.60). This suggests that participants assume that headlines labeled as AI-generated have been massively altered by AI (such as in the Strong Condition) rather than weakly altered (such as in the Weak and Medium Conditions). In other words, people assume that when a headline is labeled as AI-generated, AI did more than just improve the style of the text or write a first draft of the article, and instead did things like writing the whole article.

These findings are robust across countries (see Fig. S10), with the exception that in the United States only the Weak definition Condition is significantly different from the No definition Condition.

**Fig. 3.**
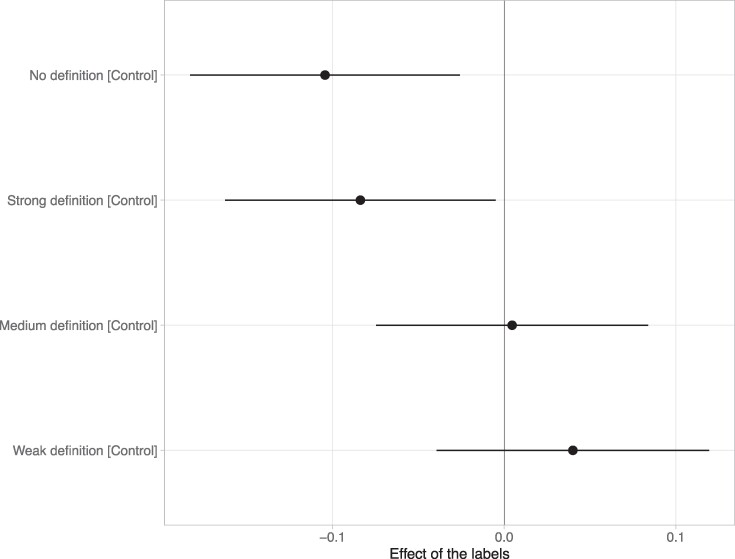
An overview of the effect of the labels on AI-generated headlines in study 2. All treatments are compared with the control in which AI-generated headlines were not labeled.

In Fig. 3, we show the effect of the labels in the treatments compared with the Control. We see that the labels have similar effects when participants were given no definitions of AI-generated headlines (*b* = −0.10 [−0.18, −0.03], *P* = 0.009) and when they were told that AI-generated headlines have been entirely written by AI (*b* = −0.08 [−0.16, −0.01], *P* = 0.037). But the labels had no statistically significant effects when participants were given weak and medium definitions of AI-generated headlines (*ps* = 0.91 and 0.32).

#### How do participants explicitly understand what it means for a headline to be AI-generate (RQ_2_) and what kinds of AI uses do participants think should be labeled? (RQ_3_)

Here, we focus on conditions in which participants were not given definitions of AI-generated (i.e. the Control and No definition conditions). The most agreed-upon characteristics of a headline labeled as AI-generated were that AI wrote the whole article (*M* = 5.11 [1.44] on the 7-point scale), while the least agreed-upon characteristics were that AI selected the topic of the article (*M* = 3.29 [1.68]) and that AI fact-checked the content of the article (*M* = 3.63 [1.69]). Participants also agreed that other characteristics, such as summarizing an article to create a headline or writing a first draft of the article, counted as AI-generated (*M* = 4.77 [1.44]). In Fig. S11, we report the full results.

The most agreed-upon use of AI that participants thought deserved a label was AI writing the whole article (*M* = 5.77 [1.62] on the 7-point scale). Participants were also in favor of labeling most other AI uses, although these preferences were weaker (*M* = 5.09 [1.68]). In Fig. S12, we report the full results. Overall, the findings of RQ_2_ and RQ_3_ suggest that the differences between the Strong and Medium Conditions observed in the experiments are due to the fact that in the Strong Condition, AI wrote the whole text, whereas in the Medium Condition, AI only wrote the first draft (and not because of differences in topic selection). Moreover, they support the conclusion that without explanations about the meaning of the labels, participants assume full AI automation.

In Supplementary material, we report exploratory analyses on differences across conditions. In short, we found that participants’ understanding of what it means for a headline to be AI-generated is in accordance with the definitions they were given in the treatment. For instance, participants in the Weak Condition were more likely to think AI is used to clarify or improve the text, whereas participants in the Strong Condition were more likely to think that AI is used to write full articles. Moreover, participants were more likely to report that labels should be applied to AI uses that fall within the definitions they were given in the treatment.

#### Estimating spillover effects of the labels on unlabeled headlines (RQ_4_)

To test RQ_4_, we compared ratings of unlabeled headlines in the Control *vs.* No definition Condition. Just like in Study 1, we found that the presence of AI-generated labels had no statistically significant effect on unlabeled headlines (*b* = 0.04 [−0.04, 0.11], *P* = 0.34).

## Discussion

We found that labeling headlines as AI-generated reduced the perceived accuracy of the headlines and participants’ intention to share them. This effect was not moderated by the origin of the headlines (human- *vs.* AI-generated) or the veracity of the headlines (true *vs.* false). Human-generated headlines labeled as AI-generated suffered from the same decrease in perceived accuracy and sharing intentions as AI-generated headlines labeled as AI-generated.

The sizes of our treatment effects are similar to those observed in past studies on human-generated headlines and AI-generated headlines. First, Longoni et al. (15) found that labeling human-generated headlines as AI-generated, compared with labeling human-generated headlines as human-generated, reduced the perceived accuracy of the headlines by 1.9pp when participants were only exposed to one kind of label (i.e. only AI labels or only human labels) and 3.63pp when participants were exposed to both kinds of labels. We found that, on average, human-generated headlines labeled as AI-generated suffered from a 2.11pp decrease in perceived accuracy and a 3.61pp decrease in sharing intentions compared with nonlabeled human headlines. These similarities in effect size, although we did not label human headlines as human-generated, support the idea that, by default, people expect nonlabeled headlines to be human-generated.

Second, Toff and Simon (16) found that labeling AI-generated articles, compared with not labeling them, reduced the perceived trustworthiness of the articles by 3.63pp. We found that AI-generated headlines labeled as AI-generated suffered from a similar decrease in perceived accuracy (3.66pp in Study 1 and 2pp in Study 2). It is thus likely that the effect of the labels is not specific to news headlines and generalizes to news articles.

Both Longoni et al. (15) and Toff and Simon (16) explained to participants that AI-generated meant that the AI was fully autonomous: “a fully autonomous Artificial Intelligence (AI) content engine to identify, curate, and produce newsworthy stories, creating content without any human prompting required” (16) and “algorithmic processes that convert data into narrative news texts with limited to no human intervention” (15). Study 2 demonstrates that by default, in the absence of definitions for “AI-generated,” people assume full AI automation (such as AI writing the whole article). It is only when given weaker, and arguably more realistic definitions (such as AI writing a draft or improving the clarity and style of the text), that the negative effects of labeling headlines as AI-generated disappear. Conversely, Study 2 demonstrates that the negative effect of “AI-generated” labels on the perceived accuracy of headlines and participants’ willingness to share them derives from maximal and possibly unrealistic assumptions regarding the meaning of the labels.

Finally, small changes in the wording of the labels are unlikely to be very consequential given that we used different wording in Studies 1 and 2 (“Article generated by AI” *vs. “*Text generated by AI”) and that very different labels have yielded similar effect sizes–e.g. “written by an AI reporter” (15).

The effects of AI-generated labels are small, especially compared with the effect of false labels. In Study 1, we found that the effect of labeling headlines as AI-generated (2.66pp) was three times smaller than the effect of labeling headlines as false (9.33pp). This is in line with recent evidence showing that US participants report that they would feel more negatively about someone posting content labeled as “Manipulated” or “Not real” than “AI-Generated” (19). It suggests that people do not equate “AI-generated” with “False” but are nonetheless more suspicious of content labeled as AI-generated than nonlabeled content. This form of AI aversion is well-documented in the literature (18).

In both studies, we found that the effects of the labels on the perceived accuracy of the headlines tended to be more robust than the effect on sharing intentions—especially in Study 1 (see also: 17). While this could mean that AI-generated content may spread despite being perceived as inaccurate, it may simply reflect the fact that people are unwilling to share news online (especially when political), regardless of whether it is AI or human-generated. Indeed, the discrepancy between accuracy and sharing may come down to a floor effect in the sharing condition. In Study 1, 6.7% reported being “Extremely unlikely” to share all headlines while only 0.05% of participants rated all headlines as “Certainly false.” Overall, sharing intentions were much lower (*M* = 2.29, median = Unlikely to share) than accuracy ratings (*M* = 3.50, median = Possibly true). This is in line with data showing that people rarely share news online and largely avoid sharing anything political (25).

A limitation of the present findings is that they are restricted to the narrow US and UK contexts. For instance, Epstein et al. (19) found that while US participants reported that they would feel negatively about people sharing and posting AI-generated content, participants in Mexico and Brazil reported that they would feel positively (although Brazilian participants also reported that it would be appropriate to classify misleading content as AI-generated). Moreover, our findings are limited to headlines and may not generalize to all types of content on social media such as videos or images. In Study 2, we manipulate different dimensions of AI definitions and do not know which specific dimension caused the treatment effect. In particular, we do not know if it is the fact that AI selected the topic, that it wrote the whole article, or both, that induced participants’ skepticism in the Strong Condition. Results from the self-reported questions suggest that it may be AI writing the full article, rather than AI selecting the topic of the article, that induced participants’ skepticism. Future research, as well as news organizations or platforms that intend on using labels for such strong AI uses, could conduct experiments to causally unbundle this treatment effect.

Despite these limitations, our experiments allow for causal inferences to be made about the effect of labeling headlines as AI-generated, and the mechanisms responsible for this AI aversion, with clear practical implications for platforms and policymakers. In both studies, we found that labeling content as AI-generated does not significantly affect unlabeled content, trust in media, or concerns about AI among respondents. This indicates that the effects of the labels are confined to labeled content. However, we do observe unintended negative consequences on both perceived accuracy and sharing intentions of the headlines—even when the content is factual and human-generated but incorrectly labeled as AI-generated. We show that this AI aversion is due to expectations that headlines labeled as AI have been fully generated by AI. It suggests that the use of AI labels should come with clear, easy-to-understand information about what the labels mean, otherwise people will assume extreme scenarios. Current labels implemented by social media platforms are vague (e.g. “Creator labeled as AI-generated” or “AI info”) and likely to be understood by people as meaning that the content has been fully AI-generated with no human supervision, even though such fully automated AI uses are uncommon, especially in journalism (24).

The effect of the labels depends on how people perceive them and react to them. While we found that people use such labels as a heuristic to infer that a headline is inaccurate (26)—just like people use brand, content, social, or platform cues as shortcuts to determine what news to trust online (27)—this heuristic may change over time. For instance, if in the next few years AI is used mostly to spread misleading content, the effect of the labels on perceived accuracy may intensify, as the associations people make between AI and inaccurate will strengthen. The effect on sharing intentions may intensify as well, as social norms around the sharing of AI content may become more stringent, with people condemning the sharing of AI content—as they do for the sharing of false news (28). Perceptions that drive heuristics may not only be affected by people's experience with AI content but also by narratives about AI and how AI is covered in the news. If people mostly hear about the potential of AI to create and disseminate false news, they may grow more skeptical of AI. On the other hand, if the use of AI for content generation becomes normalized and accepted, with a balanced understanding that AI can be used to create both useful and harmful content, the effect of labels may weaken over time. While the specific ways in which perceptions of AI will change are hard to predict, and depend on how labels will be used, a clear understanding of how they are perceived now offers an important foundation.

The utility of AI labels warrants careful consideration. The primary concern seems to be that generative AI could accelerate the creation and dissemination of harmful content, such as misinformation (10). However, it is crucial to differentiate between the origin of the content and its harmfulness. If the content is harmful, it should be moderated, regardless of whether it is AI-generated or human-generated. If the content is not harmful, the benefits of labeling it as AI-generated are debatable, especially given the negative effects of these labels. For instance, reducing the spread of factual AI-generated headlines such as the ones used in the present experiment would be detrimental to society.

We show that the effect of labeling false AI-generated headlines as AI-generated is three times smaller than the effect of labeling it as false. Thus, when it comes to false AI-generated headlines, it would be much more effective to label them as false rather than solely as AI-generated. Future studies should investigate whether it is also the case for other kinds of harmful content (such as hate-speech or nonconsensual pornography, which accounts for a large share of online deepfakes; 29) and whether combining labels enhances their effectiveness (e.g. using false and AI-generated labels) or whether it confuses people.

Additionally, labeling content as AI-generated presents challenges related to detection, beginning with defining what constitutes AI-generated content. Various degrees of human oversight can exist in the creation process, complicating the criteria for labeling. Even if a consensus on definition was reached, technical challenges in accurate detection remain significant. Given the lack of conclusive evidence that AI-generated content is inherently more likely to be harmful, our findings suggest that it may be more productive to focus on harmfulness directly rather than on the content's origin. At the same time, undisclosed AI uses by news outlets may have reputational costs for individual news organizations as well as, potentially, the industry at large. This concern highlights the need for clearer understanding and expectations regarding the content that deserves to be labeled as AI (where is the threshold, in terms of AI vs. human involvement in content creation?) as well as the reasons for doing so (what are the harms linked to AI content creation that justifies labeling?). Clarifying these questions, and informing the public, is an important task to which journalists can contribute, as well as researchers and other actors. Our study shows that a simplistic use of AI labels could be counterproductive and invites a deeper reflection on the appropriate use of labels for AI-generated content.

## Supplementary Material

pgae403_Supplementary_Data

## Data Availability

The preregistration, data, R scripts, and materials necessary to reproduce the results and the experiments are available at: https://osf.io/a4y7s/? view_only=b0961e720d7b4046bf275039119cdec3

## Notes

a More information about the preregistered hypotheses and research questions can be found in the hypotheses section. We strictly followed the analysis plan and preregistered statistical analyses and only made cosmetic changes—such as changing the wording and ordering of the hypotheses. We detail all these changes in Supplementary material. In Supplementary material, we provide more information about our design, hypotheses, and statistical analyses.
