# Supplementary material for: People are skeptical of headlines labeled as AI-generated, even if true or human-made, because they assume full AI automation
Source: PNAS Nexus. 2024 Oct 1;3(10):pgae403. doi: 10.1093/pnasnexus/pgae403 (PMC11443540; doi:10.1093/pnasnexus/pgae403)
Supplement: pgae403_Supplementary_Data [file pgae403_supplementary_data.pdf]

Appendix of Study 1

1. Treatment effects divided by sharing and accuracy ratings

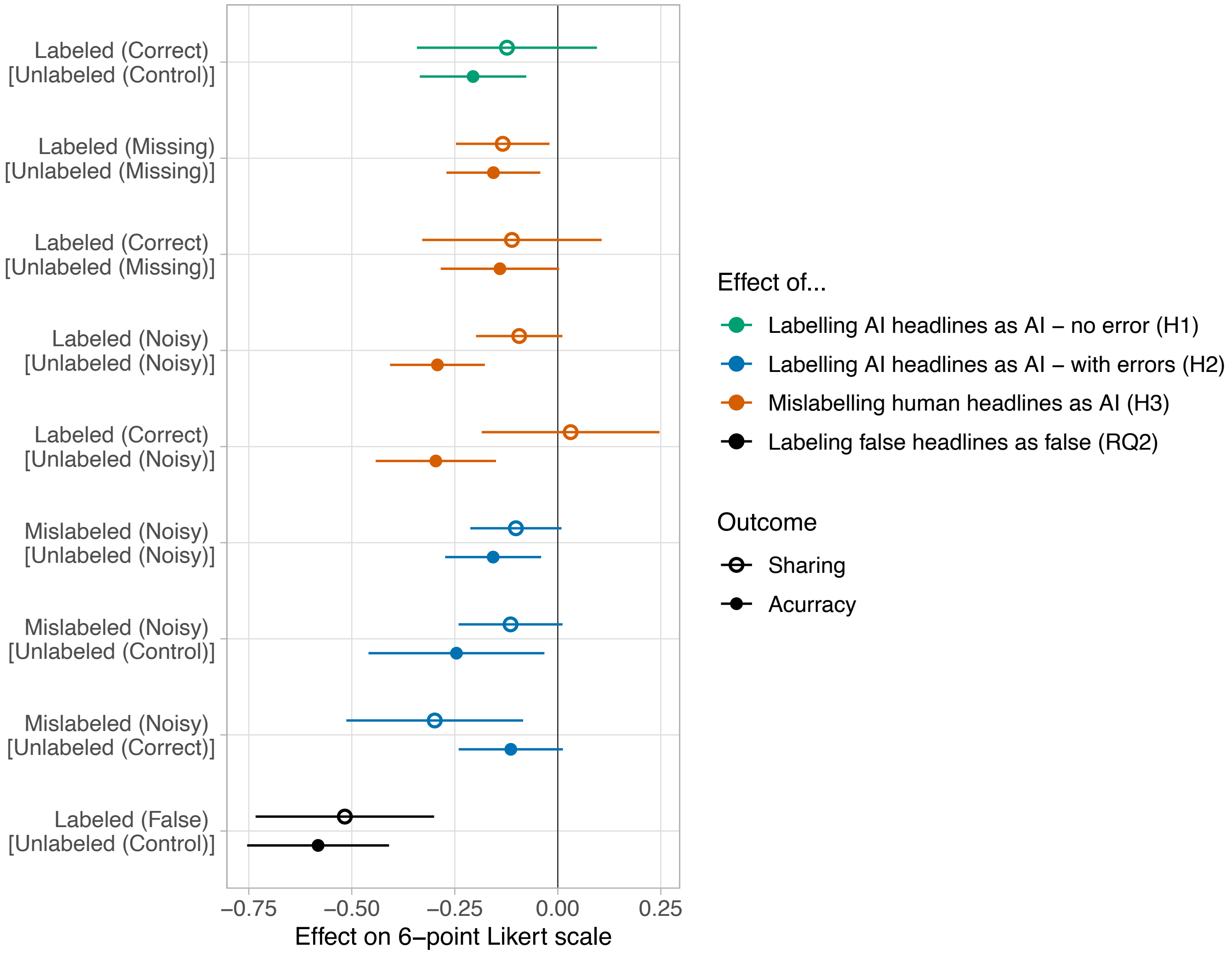

Figure S1. Treatment effects by outcome (sharing intentions *versus* perceived accuracy).

2. Treatment effects by news veracity

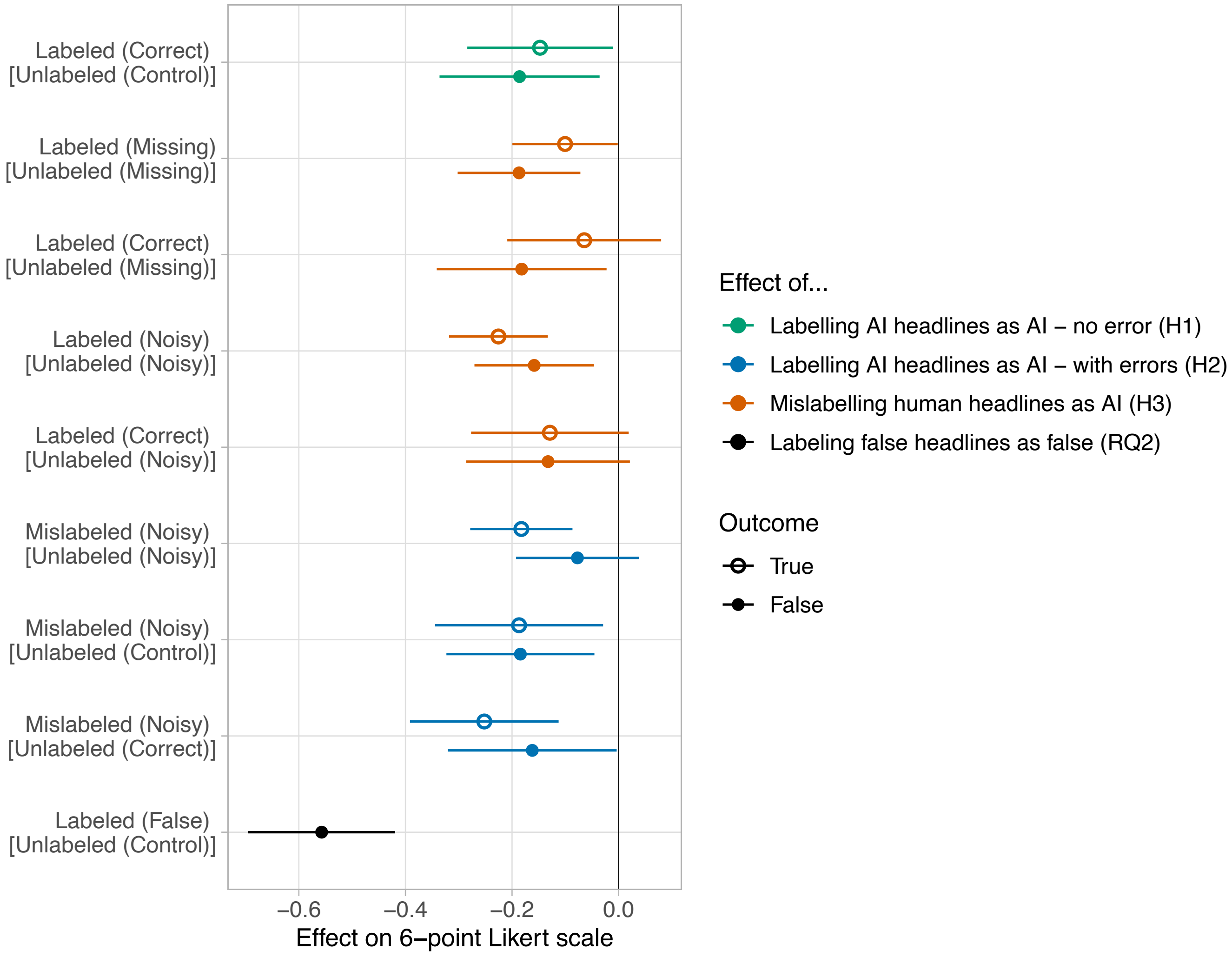

Figure S2. Treatment effects by news veracity (true *versus* false).

3. Spillover effect of the labels on unlabeled news.

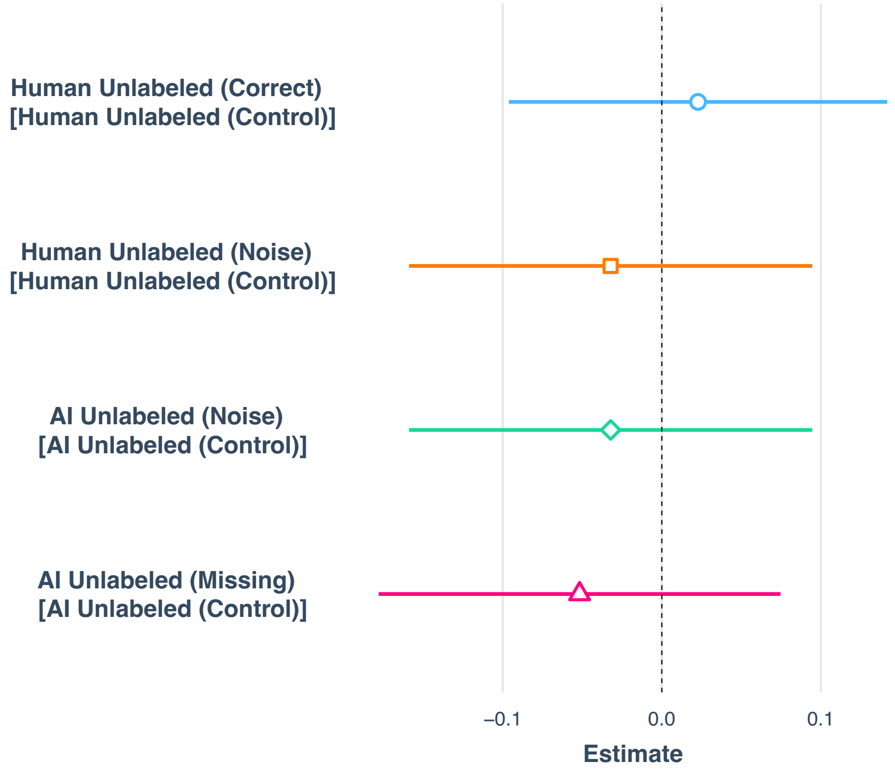

Figure S3. Effect of the presence of labels on unlabeled AI-generated and human-generated headlines in the treatments compared to (unlabeled headlines) in the Control Condition.

4. Spillover effect of the labels on trust in the news and journalists.

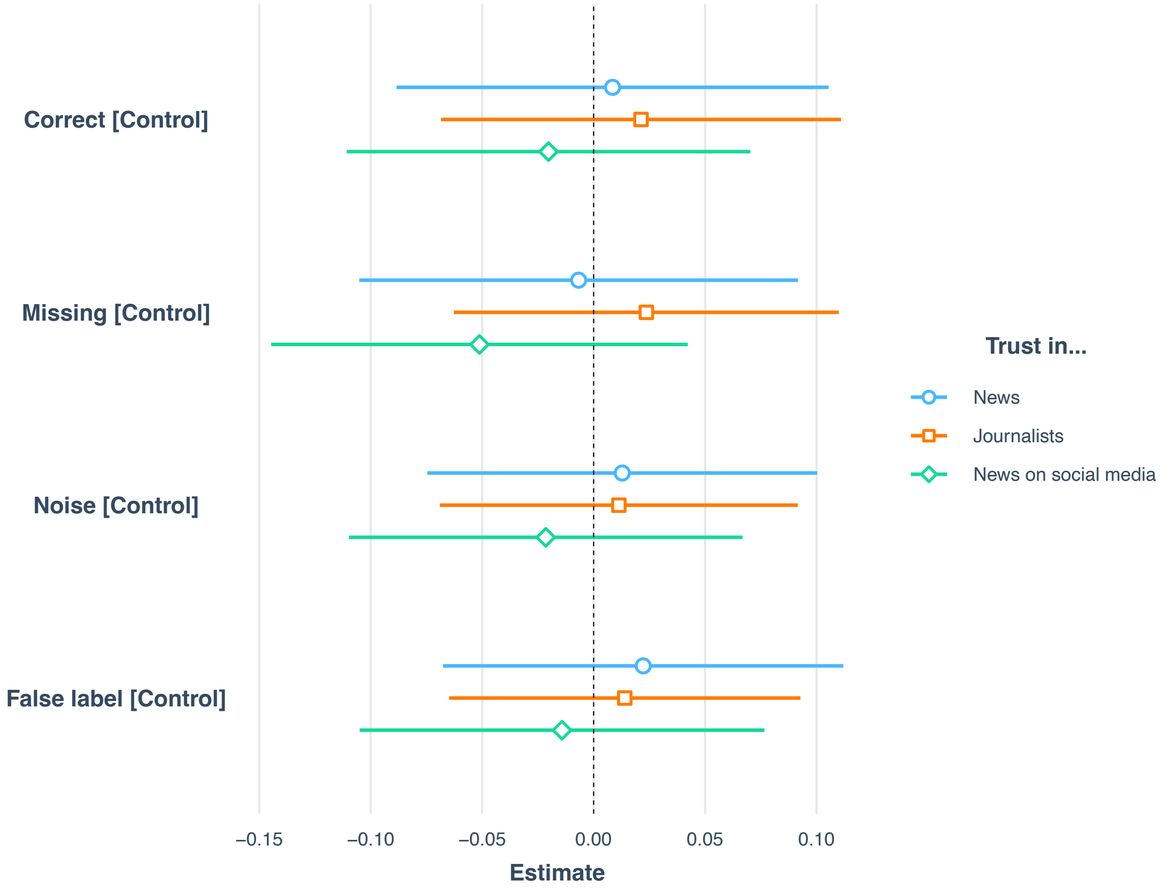

Figure S4. Effect of the presence of labels on trust in the news and journalists.

5. Spillover effect of the labels on attitudes towards AI.

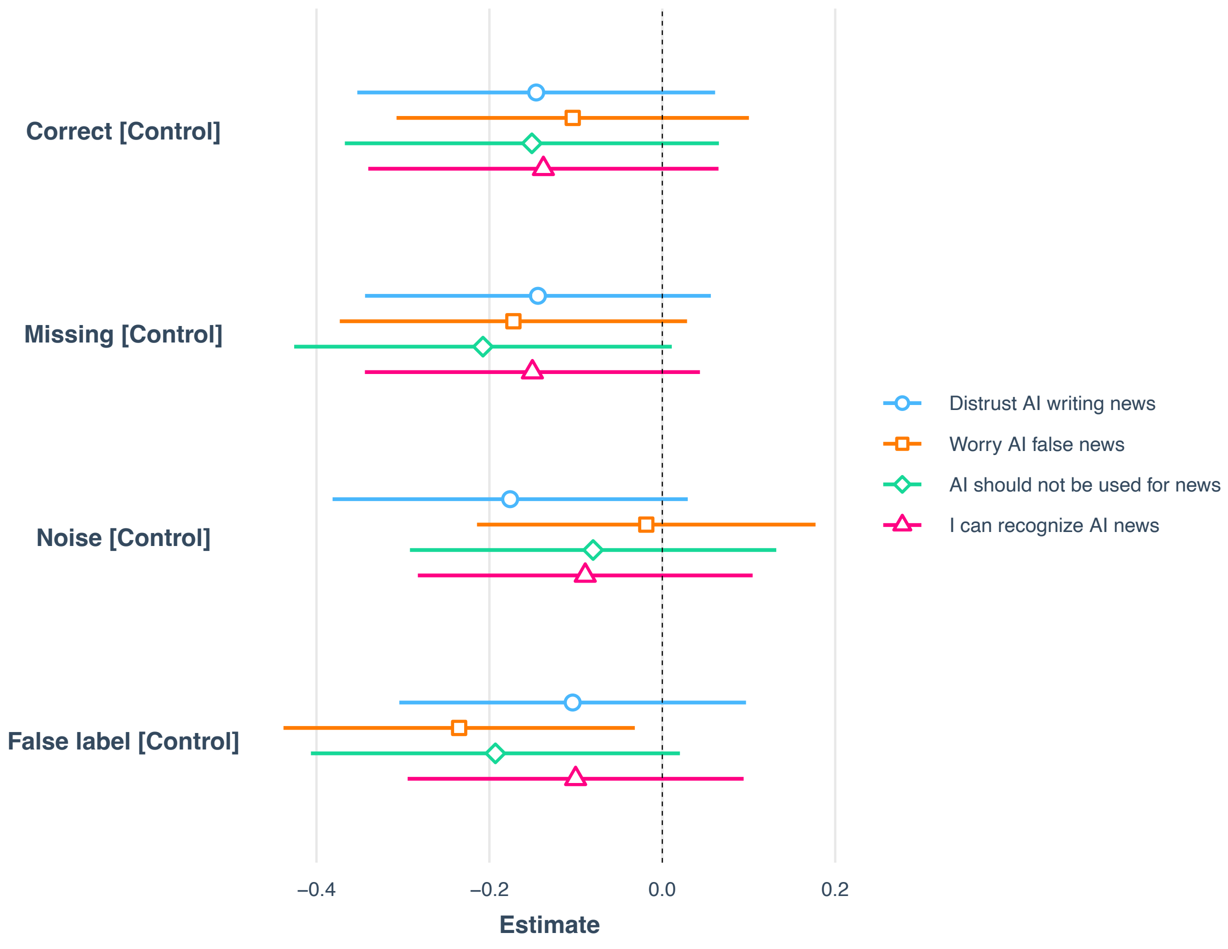

Figure S5. Effect of the presence of labels on attitudes towards AI.

6. Spillover effect of the labels on attitudes towards labeling.

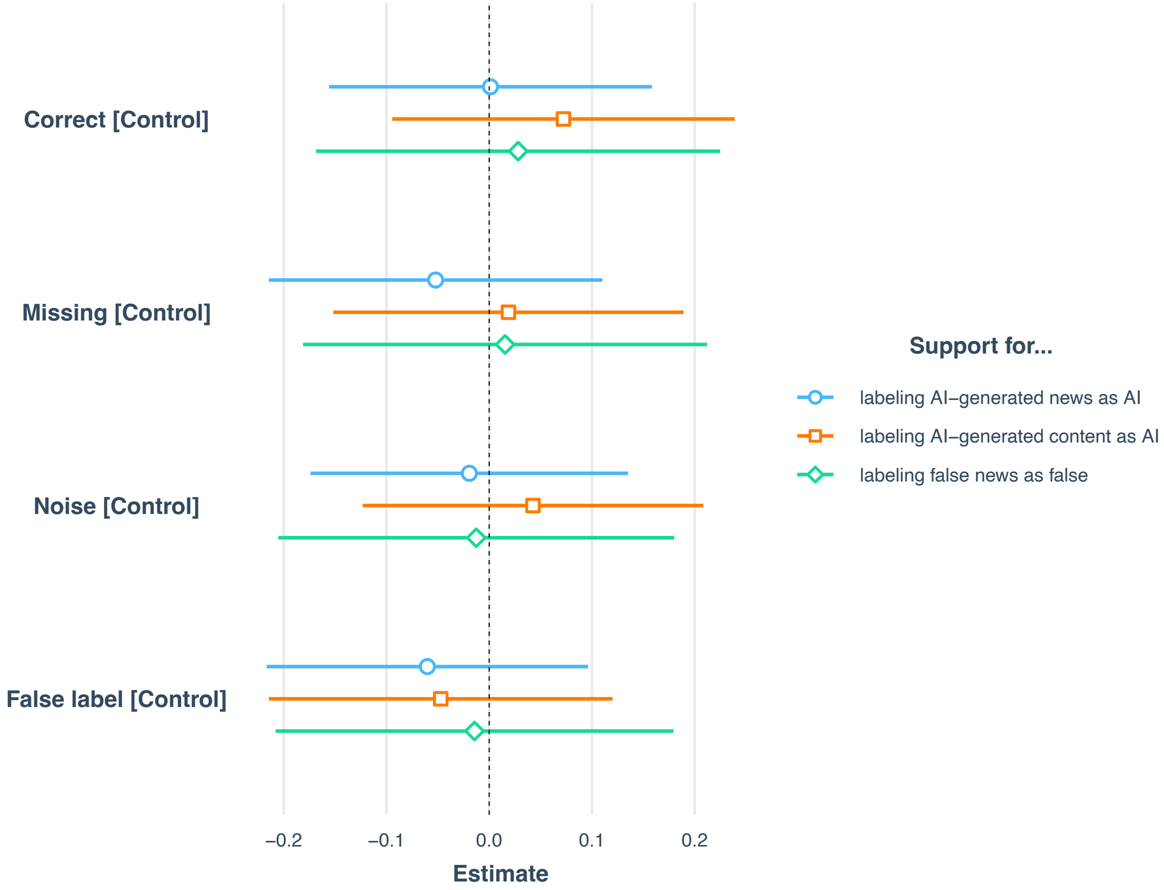

Figure S6. Effect of the presence of labels on attitudes towards labeling.

7. Perceived effect of the labels

After reporting their attitudes towards AI, participants reported the anticipated effect that labeling headlines as AI-generated would have on the perceived accuracy of the headlines and their willingness to share the headlines. Participants were told "Imagine you see a headline on social media with a label indicating that the headline has been 'Generated by Artificial Intelligence'". They were then asked to answer two questions, first "Would the label influence whether you think the events described in the headline actually occurred?" (from "Makes me more likely to think it's FALSE" [1] to "Makes me more likely to think it's FALSE" [5], with "It would have no effect" [3] as the middle point) and second "Would the label influence your decision to share the headline?" (from "Makes me less likely to share" [1] to "Makes me more likely to share" [5] with "It would have no effect" [3] as the middle point).

We found that most participants estimated that labeling headlines as AI-generated would reduce the perceived accuracy of the headline and their willingness to share them (even among participants not exposed to the labels). This contrast with Epstein et al, (2023) who found that participants estimated that the labels would have null effect on the perceived accuracy of the labeled content. This difference may be due to the nature of the stimuli: we relied on news headlines whereas they relied on images and videos–although one could expect that images and videos generated by AI are more likely to not represent reality compared to news headlines.

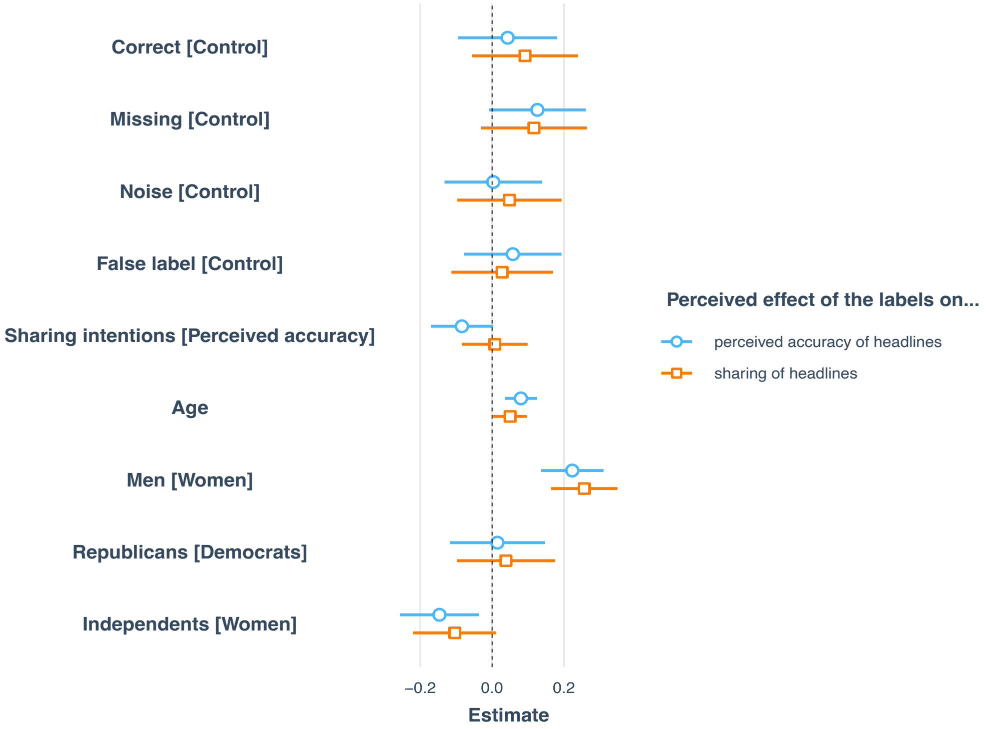

Figure S7. Predictors of perceived effect the labels would have on sharing intentions and perceived accuracy of the headlines (post-treatment).

8. Statistical models

8.1. H1

	H1 acc. & shar.	H1 (Sharing)	H1 (Accuracy)
(Intercept)	3.15 ***	2.00 ***	3.00 ***
	(0.14)	(0.24)	(0.15)
Correct [Control]	-0.17 *	-0,12	-0.21 **
	(0.06)	(0.11)	(0.07)
Sharing [Accuracy]	-1.24 ***		
	(0.06)		
True [False]	0.57 ***	-0,06	1.19 ***
	(0.03)	(0.04)	(0.04)
Age	0.01 *	0,01	0,00
	(0.00)	(0.00)	(0.00)
Men [Women]	0.13 *	0.30 **	-0,03
	(0.07)	(0.11)	(0.07)
Independent [Democrat]	-0,09	-0,10	-0,05
	(0.08)	(0.14)	(0.08)
Republican [Democrat]	0,11	0,13	0,12
	(0.09)	(0.15)	(0.10)
N	6280	3136	3144
N (PROLIFIC_PID)	785	392	393
N (News_number)	8	8	8
N (Set)	2	2	2
AIC	20975,89	10153,67	10206,42
BIC	21056,83	10220,23	10273,01
R2 (fixed)	0,19	0,02	0,19
R2 (total)	0,46	0,50	0,34

*** $p < 0.001$; ** $p < 0.01$; * $p < 0.05$.

8.2. H2

	H2a	H2b	H2c	H2d

	acc. & shar.	acc. & shar.	acc. & shar.	acc. & shar.
(Intercept)	3.03 ***	3.02 ***	3.13 ***	3.07 ***
	(0.18)	(0.15)	(0.20)	(0.15)
Missing labeled [Missing unlabeled]	-0.14 ***			
	(0.04)			
Correct labeled [Missing unlabeled]		-0,12		
		(0.07)		
Noisy labeled [Noisy unlabeled]			-0.19 ***	
			(0.04)	
Correct labeled [Noisy unlabeled]				-0,13
				(0.07)
Sharing [Accuracy]	-1.24 ***	-1.22 ***	-1.45 ***	-1.36 ***
	(0.08)	(0.07)	(0.09)	(0.07)
True [False]	0.52 ***	0.54 ***	0.54 ***	0.58 ***
	(0.04)	(0.03)	(0.04)	(0.03)
Age	0,00	0,00	0,00	0.01 *
	(0.00)	(0.00)	(0.00)	(0.00)
Men [Women]	0.21 *	0.14 *	0,02	0,05
	(0.08)	(0.07)	(0.09)	(0.07)
Independent [Democrat]	-0,01	-0,12	-0,03	-0,10
	(0.10)	(0.08)	(0.11)	(0.08)
Republican [Democrat]	0.42 ***	0,17	0,21	0,15
	(0.12)	(0.10)	(0.12)	(0.09)
N	3120	4680	3160	4700
N (PROLIFIC_PID)	390	780	395	785
N (News_number)	8	8	8	8
N (Set)	2	2	2	2
AIC	10504,34	15749,77	10576,79	15714,10
BIC	10576,88	15827,19	10649,49	15791,57
R2 (fixed)	0,20	0,19	0,24	0,22
R2 (total)	0,41	0,45	0,47	0,48

*** p < 0.001; ** p < 0.01; * p < 0.05.

8.3. H3

	H3 acc. & shar.	H3 (Sharing)	H3 (Accuracy)
(Intercept)	3.21 ***	4.12 ***	3.46 ***
	(0.13)	(0.19)	(0.16)
Noise mislabeled [Noise unlabeled]	-0.13 **	-0,10	-0.16 **
	(0.04)	(0.06)	(0.06)
Noisy mislabeled [Control unlabeled]	-0.19 **	-0.25 *	-0,11
	(0.06)	(0.11)	(0.06)
Noisy mislabeled [Correct unlabeled]	-0.21 **	-0.30 **	-0,11
	(0.06)	(0.11)	(0.06)
Sharing [Accuracy]	1.13 ***		
	(0.05)		
True [False]	-0.72 ***	-0,05	-1.40 ***
	(0.03)	(0.03)	(0.03)
Age	0,00	0,00	0,00
	(0.00)	(0.00)	(0.00)
Men [Women]	-0,09	-0,17	-0,02
	(0.05)	(0.09)	(0.05)
Independent [Democrat]	0,08	0,17	-0,02
	(0.06)	(0.11)	(0.06)
Republican [Democrat]	-0.20 **	-0,19	-0.22 **
	(0.07)	(0.12)	(0.07)
N	9440	4728	4712
N (PROLIFIC_PID)	1180	591	589
N (News_number)	8	8	8
N (Set)	2	2	2
AIC	32181,12	15751,22	15355,40
BIC	32281,25	15835,22	15439,35
R2 (fixed)	0,19	0,02	0,23
R2 (total)	0,41	0,44	0,36

*** p < 0.001; ** p < 0.01; * p < 0.05.

9. Deviations from the pre-registration

We only made cosmetic changes and strictly followed the analysis plan and pre-registered statistical analyses.

We list here are all the minor deviations from the pre-registration:

- We changed the wording of all the hypotheses and research questions (while keeping their meaning constant).
- We labeled H3a, H3a', H3b, and H3b' as H2 for ease of reporting.
- We labeled H2a, H2b, and H2b' as H3 for ease of reporting.
- We added one exploratory research question (RQ3) on the implied truth effect – as specified at the end of the introduction.
- We moved a research question (initially RQ3) on the perceived effect of labels in Appendix section 7.
- We merged RQ2 into RQ1.
- We labeled RQ4 as RQ2.

In the sections below we detail all these changes and compare the wording and ordering of the hypotheses in the pre-registration to the manuscript and to the R code used to analyze the data.

A. H1 (as it appears in the manuscript)

In the preregistration, we write:

> H1 : GenAI labels will reduce accuracy and sharing ratings
>
> data_H1 = filter(data, Condition == « Control » & AI_Human == « AI » | Condition == « Correct » & AI_Human == « AI »)
>
> lmer(ratings ~ Condition + Sharing_Accuracy + True_False + pre_treatment_demographic_and_control_variables + (1|Participants) + (1|post) + (1|News_Set), data = data_H1)
>
> H1 refers to the effect of 'Condition'.

In the manuscript, we write:

H_1 : AI-generated headlines labeled as AI-generated will be rated as less accurate and be less shared than unlabeled AI-generated headlines (when all AI-generated headlines are labeled as such).

In the R script, we do:

```
data_H1x = filter(X2, Condition == "Control" & AI_Human == "AI")
data_H1xx = filter(X2,  Condition == "Correct" & AI_Human == "AI")
data_H1 = rbind(data_H1x,data_H1xx)
```

Note that this filtering is equivalent to the one specified in the pre-registration: in both case we are filtering AI-generated headlines in the control vs correct condition.

```
model_H1 = lmer(Ratings ~ condition + DV + True_False + Age +Sex +
Political_orientation + (1|News_number) + (1|Set)+ (1|PROLIFIC_PID), data =
data_H1)
```

Note that this model is equivalent to the one in the pre-registration (‘DV’ is a dummy for ‘Sharing_Accuracy’).

Thus, we did not deviate in any ways from the pre-registration, except that we changed the wording of H1.

B. H2 (as it appears in the manuscript)

In the preregistration, we write:

H3a: Unlabeled AI-generated content will receive higher accuracy and sharing ratings than labeled AI-generated content (within the Missing AI- labels Condition)

```
data_H3a = filter(data, Condition == “Missing Condition” & AI_Human == “GenAI”)
```

```
lmer(ratings ~ label + Sharing_Accuracy + True_False +
pre_treatment_demographic_and_control_variables +(1|Participants) + (1|post) +
(1|News_Set), data = data_H3a)
```

H3a’: Unlabeled AI-generated content will receive higher accuracy and sharing ratings than labeled AI-generated content (across conditions)

```
data_H3a’ = filter(data, Condition == “Missing Condition” & Type_post == “GenAI” &
unlabelled == “Yes”  | Condition == “Correct Condition” & Type_post == “GenAI” )
```

```
lmer(ratings ~ Condition + Sharing_Accuracy + True_False
+pre_treatment_demographic_and_control_variables + (1|Participants) + (1|post) +
(1|News_Set), data = data_H3a’)
```

H3b: Unlabeled AI-generated content will receive higher accuracy and sharing ratings than labeled AI-generated content (within the Noise AI- labels Condition)

data_H3a = filter(data, Condition == “Noise Condition” & Type_post == “GenAI”)

lmer(ratings ~ label + Sharing_Accuracy + True_False + pre_treatment_demographic_and_control_variables +(1|Participants) + (1|post) + (1|News_Set), data = data_H4b)

H3b’: Unlabeled AI-generated content will receive higher accuracy and sharing ratings than labeled AI-generated content (across conditions)

data_H4b’ = filter(data, Condition == “Noise Condition” & Type_post == “GenAI” & unlabelled == “Yes” | Condition == “Correct Condition” & Type_post == “GenAI”)

lmer(ratings ~ Condition + Sharing_Accuracy + True_False + pre_treatment_demographic_and_control_variables + (1|Participants) + (1|post) + (1|News_Set), data = data_H4b’)

In the manuscript, we write:

H_2 : AI-generated headlines labeled as AI-generated will be rated as less accurate and be less shared than unlabeled AI-generated headlines (when not all AI-generated headlines are labeled and when some are mislabeled).

Here, we condensed H3a, H3a’, H3b, and H3b’ into one hypothesis (H2) for ease of reporting. Yet, as it should be clear below, we tested all variants as pre-registered.

In the manuscript we flipped the wording compared to the pre-registration: instead of writing that ‘Unlabeled AI-generated content will receive higher scores’ we now write in the manuscript that ‘Labeled AI-generated content will receive lower scores’. The two framings are logically equivalent.

To test H3a the R script, we do:

data_H4a = filter(X2, Condition == "Missing" & AI_Human == "AI")
model_H4a = lmer(Ratings_reversed ~ label + DV + True_False + Age +Sex + Political_orientation+ (1|News_number) + (1|Set)+ (1|PROLIFIC_PID), data = data_H4a)

The model and filtering are equivalent to the pre-registration (although the exact specification of the filtering is different).

To test H3a’ the R script, we do:

data_H4x = filter(X2, Condition == "Missing" & AI_Human == "AI" & Label == "no")

```
data_H4xx = filter(X2,  Condition == "Correct" & AI_Human == "AI")
data_H4aa = rbind(data_H4x,data_H4xx)
data_H4aa <- mutate(data_H4aa, Condition = fct_relevel(Condition,
"Correct","Missing"))

data_H4aa$Ratings_reversed <- max(data_H4aa$Ratings) - data_H4aa$Ratings

model_H4aa = lmer(Ratings_reversed ~ Condition + DV + True_False + Age +Sex +
Political_orientation+ (1|News_number) + (1|Set)+ (1|PROLIFIC_PID), data =
data_H4aa)
```

The model and filtering are equivalent to the pre-registration (although the exact specification of the filtering is different).

To test H3b the R script, we do:

```
data_H4bb = filter(X2, Condition == "Noise" & AI_Human == "AI")

data_H4bb$Ratings_reversed <- max(data_H4bb$Ratings) - data_H4bb$Ratings
model_H4bb = lmer(Ratings_reversed ~ Label + DV + True_False + Age +Sex +
Political_orientation+ (1|News_number) + (1|Set)+ (1|PROLIFIC_PID), data =
data_H4bb)
```

The model and filtering are equivalent to the pre-registration (although the exact specification of the filtering is different).

To test H3b' the R script, we do:

```
data_H4bbx = filter(X2, Condition == "Noise" & AI_Human == "AI" & Label == "no")
data_H4bbxx = filter(X2,  Condition == "Correct" & AI_Human == "AI")
data_H4bbb = rbind(data_H4bbx,data_H4bbxx)

data_H4bbb <- mutate(data_H4bbb, Condition = fct_relevel(Condition, "Correct",
"Noise"))

model_H4bbb = lmer(Ratings_reversed ~ Condition + DV + True_False + Age +Sex +
Political_orientation+ (1|News_number) + (1|Set)+ (1|PROLIFIC_PID), data =
data_H4bbb)
```

Again, the model and filtering are equivalent to the pre-registration (although the exact specification of the filtering is different).

C. H3 (as it appears in the manuscript)

In the preregistration, we write:

H2a: Human-generated content mislabeled as GenAI will receive lower accuracy and sharing ratings than non-labeled Human-generated content (within the noise condition)

data_H2a = filter(data, Condition == “Noise” & AI_Human == “Human”)

lmer(ratings ~ label + Sharing_Accuracy + True_False + pre_treatment_demographic_and_control_variables + (1|Participants) + (1|post) + (1|News_Set), data = data_H2a)

H2a refers to the effect of ‘label’.

H2a’: Human-generated content mislabeled as GenAI will receive lower accuracy and sharing ratings than non-labeled Human-generated content (across conditions)

H2b: Human-generated content mislabeled as GenAI content will receive lower accuracy and sharing ratings than human-generated content correctly labeled.

data_H2_a’b = filter(data, Condition == “Control” & AI_Human == “Human” | Condition == “Noise” & Incorrectly_labeled == “Yes” & AI_Human == “Human” | Condition == “Correct” & AI_Human == “Human”)

lmer(ratings ~ Condition(baseline = Noise_mislabelled) + Sharing_Accuracy + True_False +pre_treatment_demographic_and_control_variables + (1|Participants) + (1|post) + (1|News_Set), data = data_H2_a’b)

In the manuscript, we write:

H_3 : Human-generated headlines labeled as AI-generated will be rated as less accurate and be less shared than human-generated headlines not labeled.

Here, we condensed H2a, H2b, and H2b’ into one hypothesis (H3) for ease of reporting. Yet, as it should be clear below, we tested all variants as pre-registered.

To test H2a, H2b, and H2b’, in the R script we do:

```
X2$Human_mislaballed_as_AI <- ifelse(X2$AI_Human == "human" & X2$Label == "AI", 1, 0)

X2$Condition = as.character(X2$Condition)
X2 <- X2 %>% mutate(Conditions = ifelse(Condition == "Noise" & Human_mislaballed_as_AI == 1, "Noise_mislabelled",ifelse(Condition == "Noise" & Human_mislaballed_as_AI == 0, "Noise_unlabelled",X2$Condition)))

data_H3x = filter(X2, Condition == "Control" & AI_Human == "human")
data_H3xx = filter(X2,  Condition == "Noise" & AI_Human == "human")
data_H3xxx = filter(X2,  Condition == "Correct" & AI_Human == "human")
```

data_H3ab = rbind(data_H3x,data_H3xx,data_H3xxx)

data_H3ab$Ratings_reversed <- max(data_H3ab$Ratings) - data_H3ab$Ratings

data_H3ab <- mutate(data_H3ab, Conditions = fct_relevel(Conditions, "Noise_mislabelled", "Noise_unlabelled","Control", "Correct"))

model_H3ab = lmer(Ratings_reversed ~ Conditions + DV + True_False + Age +Sex + Political_orientation+ (1|News_number) + (1|Set)+ (1|PROLIFIC_PID), data = data_H3ab)

Note that the first contrast in the model "Noise_mislabelled" *versus* "Noise_unlabelled" corresponds to H2a, the second contrast in the model "Noise_mislabelled" *versus* "Control" corresponds to H2a', while the last contrast "Noise_mislabelled" *versus* "Correct" corresponds to H2b.

The models and filtering are equivalent to the pre-registration (although the exact specification of the filtering is different).

D. H4 (as it appears in the manuscript)

In the preregistration, we write:

H4: GenAI labels will reduce trust in the news and journalists

lm(trust_news_post ~ trust_news_pre + Condition(baseline = control), data = all_data)

lm(trust_news_SM_post ~ trust_news_SM_pre + Condition(baseline = control), data = all_data)

lm(trust_journalists_post ~ trust_journalists_pre + Condition(baseline = control), data = all_data)

In the manuscript, we write:

H_4 : Participants exposed to headlines labeled as AI-generated will report lower trust in the news and journalists compared to participants in the Control Condition not exposed to any label.

We changed the wording of H4 to be more specific, but the meaning of the hypothesis remains unchanged.

In the R script, we do:

model_H1_news = lm(Trust_news_post ~ Trust_news_pre + Condition + Age +Sex + Political_orientation, data = X2_Wide_cut)

model_H1_journalist = lm(Trust_journalist_post ~ Trust_journalist_pre + Condition+ Age +Sex + Political_orientation, data = X2_Wide_cut)

model_H1_newsSM = lm(Trust_news_SM_post ~ Trust_news_SM_pre + Condition+ Age +Sex + Political_orientation, data = X2_Wide_cut)

The models are equivalent to the ones in the pre-registration (it's not specified here but we do take the control condition as the baseline of 'Condition').

In the pre-registration we did specify that we would include the demographic variables in the model (whereas we do in the manuscript). The results of H4 remain unchanged when removing the demographic variables from the models (i.e., all estimates remain very far from being statistically significant). We included the demographic variables in the model because when running the analyses we did not find any good reason to include them for H1-2-3 but not for H4.

E. RQ1 (as it appears in the manuscript)

In the preregistration, we write:

RQ1: What will be the effect of labeling GenAI content on perceptions of risk/benefits of AI?

lm(perception_AI_A ~ Condition(baseline = control) + pre_treatment_demographic_and_control_variables, data = all_data)

lm(perception_AI_B ~ Condition(baseline = control) + pre_treatment_demographic_and_control_variables, data = all_data)

lm(perception_AI_C ~ Condition(baseline = control) + pre_treatment_demographic_and_control_variables, data = all_data)

lm(perception_AI_D ~ Condition(baseline = control) + pre_treatment_demographic_and_control_variables, data = all_data)

RQ2: What is the effect of labeling GenAI content on perceptions of labeling?

lm(perception_labeling_A ~ Condition(baseline = control) + pre_treatment_demographic_and_control_variables, data = all_data)

lm(perception_labeling_B ~ Condition(baseline = control) + pre_treatment_demographic_and_control_variables, data = all_data)

lm(perception_labeling_C ~ Condition(baseline = control) + pre_treatment_demographic_and_control_variables, data = all_data)

In the manuscript, we write:

> RQ_1: What is the effect of being exposed to headlines labeled as AI-generated on attitudes toward AI, the news, and support for labeling?

Note that we combined RQ1 and RQ2, and changed the wording to be more precise.

In the R script, we do:

```
model_RQ1_1 = lm(distrust_AI_writing_accurate_news ~ Condition + Age +Sex +
Political_orientation, data = X2_Wide_cut)

model_RQ1_2 = lm(worry_AI_false_news ~ Condition+ Age +Sex +
Political_orientation , data = X2_Wide_cut)

model_RQ1_3 = lm(news_shouldnt_use_AI ~ Condition+ Age +Sex +
Political_orientation , data = X2_Wide_cut)

model_RQ1_4 = lm(ability_to_tell_AI_news ~ Condition + Age +Sex +
Political_orientation, data = X2_Wide_cut)
```

These models allow us to test RQ1, as specified in the pre-registration.

```
model_RQ2_1 = lm(support_label_AI_news ~ Condition + Age +Sex +
Political_orientation, data = X2_Wide_cut)

model_RQ2_2 = lm(support_label_AI_general ~ Condition+ Age +Sex +
Political_orientation , data = X2_Wide_cut)

model_RQ2_3 = lm(support_label_false ~ Condition + Age +Sex +
Political_orientation, data = X2_Wide_cut)
```

These allow us to test RQ2, as specified in the pre-registration.

F. RQ2 (as it appears in the manuscript)

In the preregistration, we write:

> RQ4: Is the effect of the genAI label similar to the effect of a false label?

```
data_RQ5 = filter(data, Condition == “Correct AI-labels Condition” & Type_post ==
“GenAI” & veracity == “false”| Condition == “Veracity-labels Condition” & Type_post
== “GenAI” & veracity == “false”)
```

```
lmer(ratings ~ Condition + sharing vs belief +
pre_treatment_demographic_and_control_variables + (1|Participants) + (1|post),
data = data_RQ5)
```

In the manuscript, we write:

> RQ_2: Is the effect of AI-generated labels on accuracy ratings and sharing intentions similar to the effect of false labels?

We changed RQ4 into RQ2.

In the R script, we do:

```
data_RQ4x = filter(X2, Condition == "Correct" & AI_Human == "AI" & True_False ==
"false")
data_RQ4xx = filter(X2,  Condition == "FalseLabel" & AI_Human == "AI"& True_False
== "false")
data_RQ4 = rbind(data_RQ4x,data_RQ4xx)

model_RQ4 = lmer(Ratings ~ Condition + DV  + Age +Sex + Political_orientation+
(1|News_number) + (1|Set)+ (1|PROLIFIC_PID), data = data_RQ4)

data_RQ4_sha = filter(data_RQ4, DV == "Sharing")
```

This is the pre-registered model that we report in the manuscript.

In the manuscript we also conducted this exploratory model comparing the false label to the control condition:

```
model_RQ4 = lmer(Ratings ~ Condition   + Age +Sex + Political_orientation+
(1|News_number) + (1|Set)+ (1|PROLIFIC_PID), data = data_RQ4_sha)
summ(model_RQ4, confint =T)

data_RQ4x = filter(X2, Condition == "Control" & True_False == "false")
data_RQ4xx = filter(X2,  Condition == "FalseLabel" &  True_False == "false")
data_RQ4 = rbind(data_RQ4x,data_RQ4xx)

model_RQ4 = lmer(Ratings ~ Condition + DV  + Age +Sex + Political_orientation+
(1|News_number) + (1|Set)+ (1|PROLIFIC_PID), data = data_RQ4)
```

10. Information about our design

A. Why having true and false news?

We included true and false news in the experiment, even though we had no hypotheses about potential differences between true and false news, because it is likely that news articles generated by AI will be both true and false. It is thus important to include both true and false news to reflect this reality. Moreover, from an applied perspective, negative effects on true and false news are not synonymous: it is commonly agreed that negative effects on true news are detrimental whereas negative effects on false news are beneficial. In this regard, this decision to include true and false news is in line with past work on the effect of gen-AI labels and labels more broadly.

B. Why having human and AI-generated news?

We included human-generated news in the experiment because we tested hypotheses about the effect of human-generated headlines mislabeled as AI-generated. We included AI-generated news in the experiment because if such labeled are applied on social media it is AI-generated news that will predominantly be labeled as such.

11. Information about our statistical analyses and hypotheses

A. Why H1?

AI-generated headlines labeled as AI-generated will be rated as less accurate and be less shared than unlabeled AI-generated headlines (when all AI-generated headlines are labeled as such).

We test H1 by comparing AI-generated headlines that are either labeled or not labeled across two conditions: the Control condition (where no headline is labeled) and the Correct condition (where all AI-generated headlines are correctly labeled as such). This is the most straightforward way to test this simple hypothesis, there are no other way to test it with our design.

B. Why H2?

AI-generated headlines labeled as AI-generated will be rated as less accurate and be less shared than unlabeled AI-generated headlines (when not all AI-generated headlines are labeled and when some are mislabeled).

H2 is a slightly different version of H1, where the effect of the labels is estimated when not all AI-generated headlines are correctly labeled. To test this hypothesis, we need conditions where some AI-generated headlines are not labeled (while others are) and where some AI-generated headlines are mislabeled (while other are correctly labeled).

Unlike H1, where the effect of the labels was estimated by taking as a reference point unlabeled AI-generated headlines in the Control condition, H2 takes as a reference unlabeled AI-generated headlines in the Missing labels condition and in the Noisy labels condition

We test H2 by conducting four contrasts:

- Unlabeled AI-generated headlines in the Missing labels condition *versus* labeled AI-generated headlines in the Missing labels condition
- Unlabeled AI-generated headlines in the Missing labels condition *versus* labeled AI-generated headlines in the Correct label condition

These contrasts allow us to estimate the effect of the labels when some AI-generated headlines, but not others, are labeled as AI-generated (thanks to the Missing labels condition).

- Unlabeled AI-generated headlines in the Noisy labels condition *versus* labeled AI-generated headlines in the Noisy labels condition
- Unlabeled AI-generated headlines in the Noisy labels condition *versus* labeled AI-generated headlines in the Correct label condition.

These contrasts allow us to estimate the effect of the labels when some AI-generated headlines, but not others, are mislabeled as AI-generated (thanks to the Noisy labels condition). There are no other ways to test this hypothesis, we test all the possible contrasts.

C. Why H3?

Human-generated headlines labeled as AI-generated will be rated as less accurate and be less shared than human-generated headlines not labeled.

H3 is a again a slightly different version of H1, where this time the effect of labels is estimated on human-generated headlines. To test this hypothesis, we need human headlines labeled as AI-generated and unlabeled human generated. We thus took as a reference point the human-generated headlines labeled as AI-generated in the Noisy Condition (the only condition in which human-generated headlines are mislabeled) and compared it to all the other unlabeled human-generated headlines (in the Noisy, Control, and Correct condition). Merging these three conditions together is possible but would ignore important differences between conditions (e.g., in the Control there are no labels while labels are present in the Correct condition).

We test H2 by conducting three contrasts:

- Human-generated headlines labeled as AI-generated in the Noisy condition *versus* unlabeled human-generated headlines in the Noisy condition.
- Human-generated headlines labeled as AI-generated in the Noisy condition *versus* unlabeled human-generated headlines in the Control condition.
- Human-generated headlines labeled as AI-generated in the Noisy condition *versus* unlabeled human-generated headlines in the Correct condition.

There are no other ways to test this hypothesis, we test all the possible contrasts.

12. Why merging sharing and accuracy ratings?

Readers may wonder why we would merge accuracy and sharing ratings in the main analyses even though these are two very different metrics.

First, our main hypotheses are not specific to 'sharing' or 'accuracy'. We use these two constructs as a way to test whether the labels negatively influence perceptions of the headlines. We could have used alternative metrics such as participants' willingness to like the headlines or comment the headlines, or we could have measured various perceptions of the headlines such as their quality or their appeal. We selected sharing and accuracy ratings because they are widely used in the literature on headlines perceptions and are the main dependent variables of studies testing the effect of labels on headlines perceptions (because sharing is a central element

of social media platforms and because accuracy perceptions are considered to be particularly important for the study of misperceptions).

Second, we pre-registered and designed the experiment to merge sharing and accuracy ratings. Participants were either asked the accuracy or the sharing question, so we have much less power to detect an effect if we measure treatment effects separately (see Figure 1 in Appendix).

Third, in the main text and in Appendix, we report all the results separately for accuracy and sharing ratings, and compare effect sizes across variables.

Appendix of Study 2

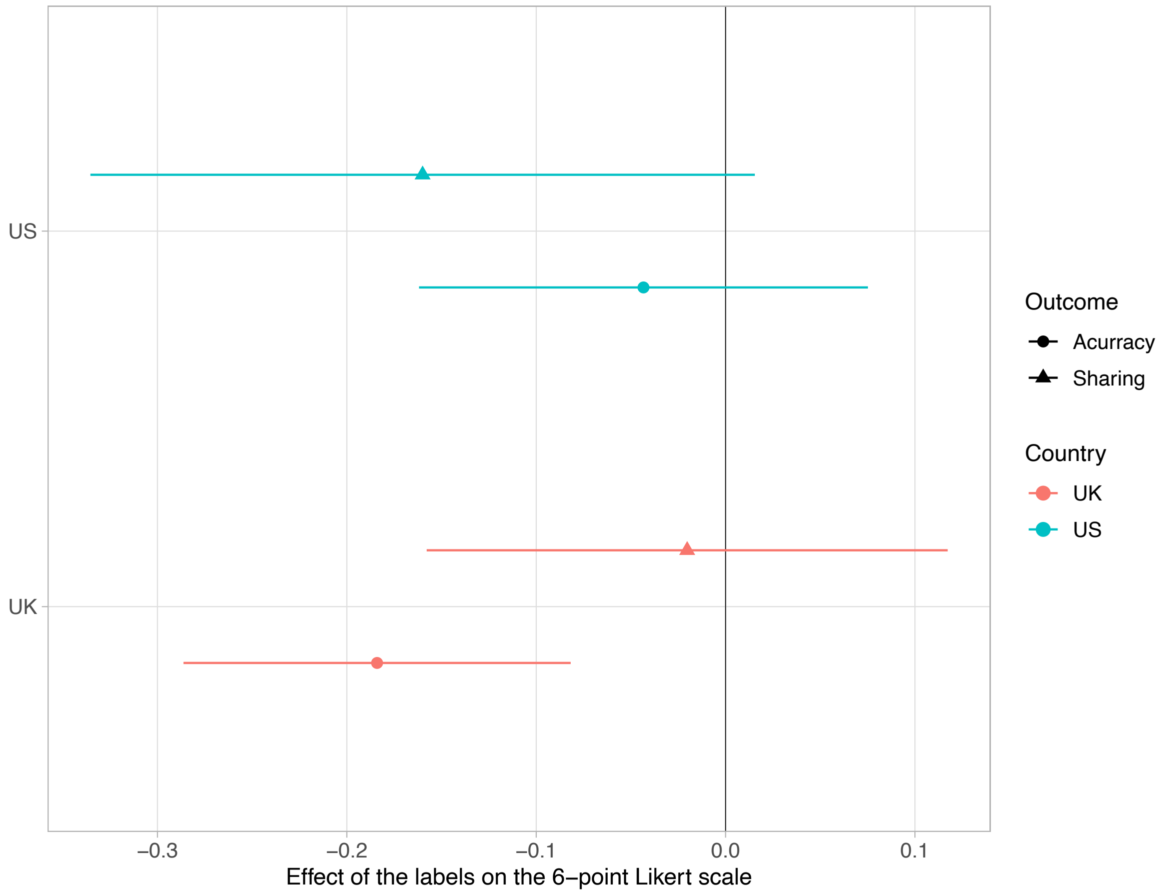

Figure S8. Effect of the labels broken down by countries and outcome measures (while controlling for veracity of the headlines, gender, age, political orientation, and with participant ID, headline, and headline set as random effect).

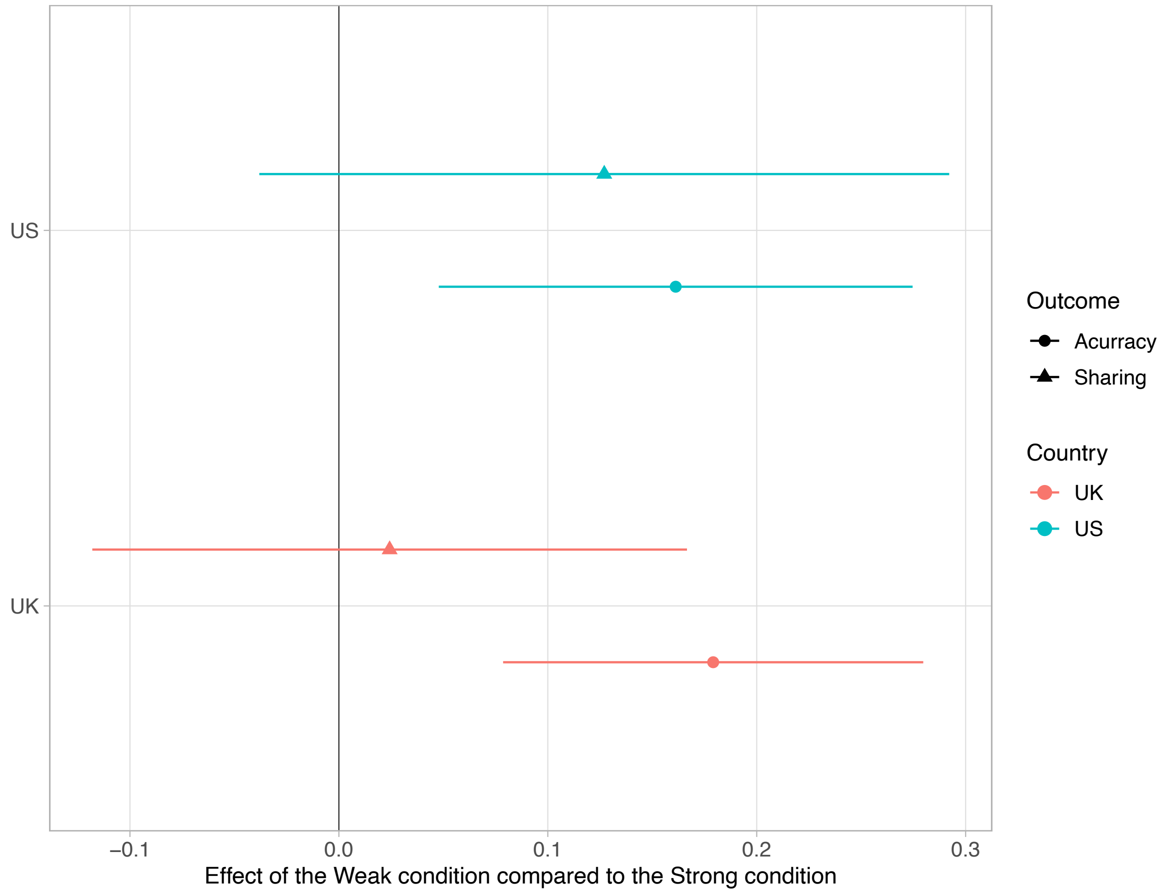

Figure S9. Effect of the labels in the Weak condition compared to the Strong condition, broken down by countries and outcome measures (while controlling for veracity of the headlines, gender, age, political orientation, and with participant ID, headline, and headline set as random effect).

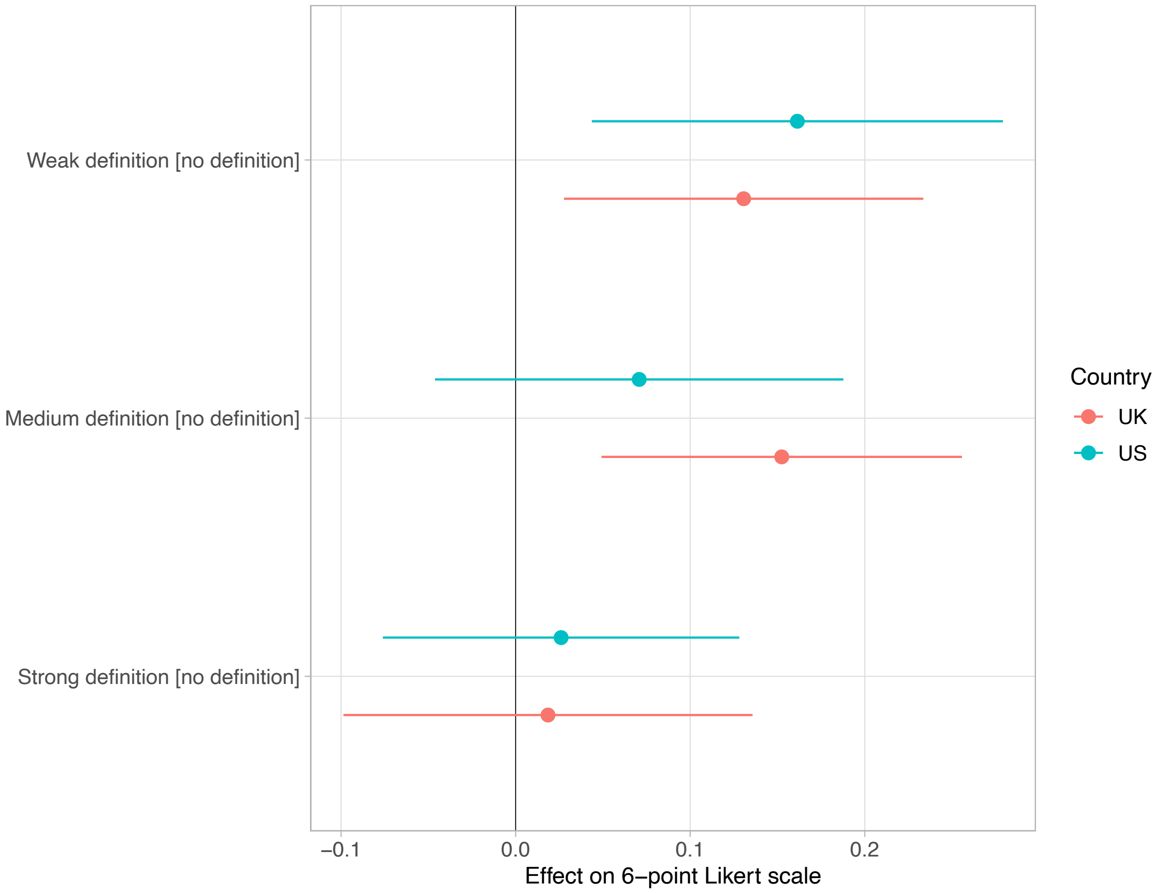

Figure S10. Effect of the labels in the Weak, Medium, and Strong Conditions compared to the No definition Condition. In all conditions the AI-generated headlines are labeled as AI-generated. The effects are broken down by countries (while controlling for veracity of the headlines, gender, age, political orientation, and with participant ID, headline, and headline set as random effect).

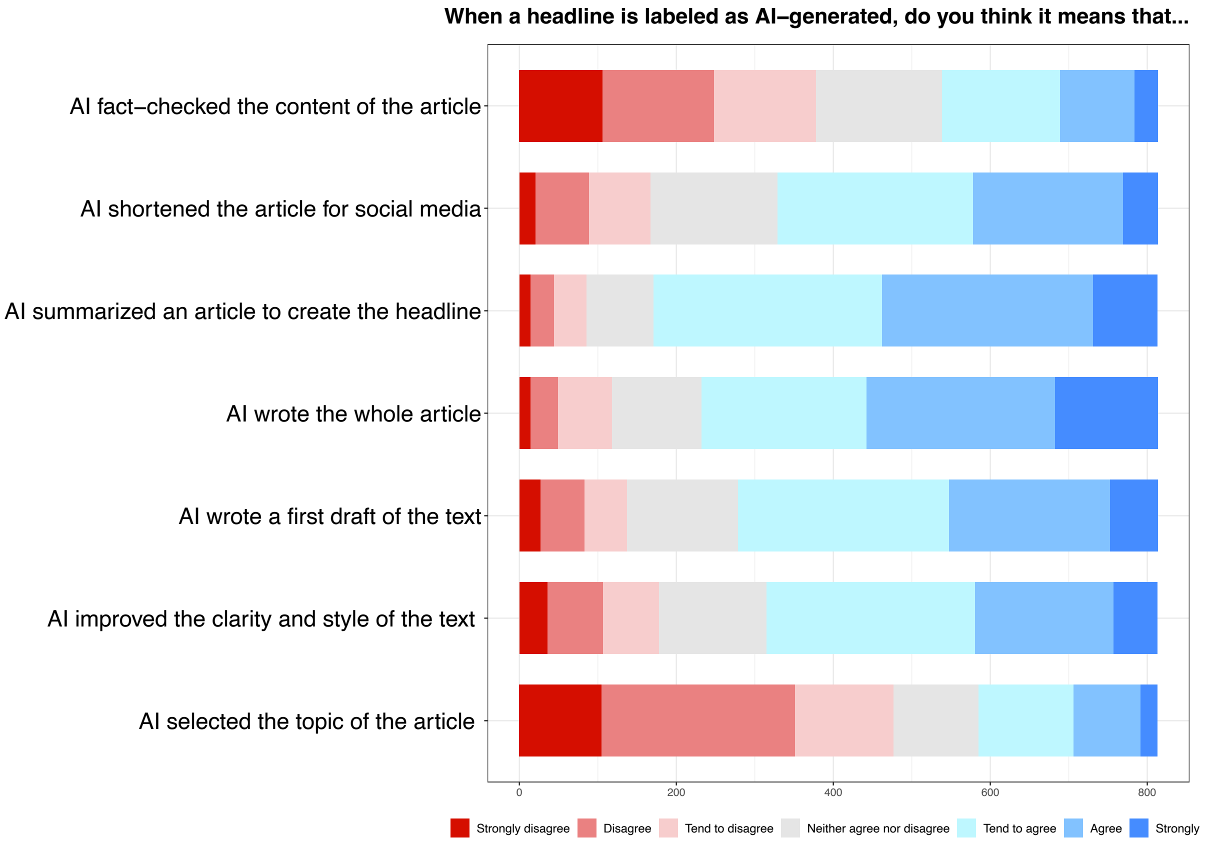

Figure S11. How do participants explicitly understand what it means for a headline to be AI-generated? (RQ_2)

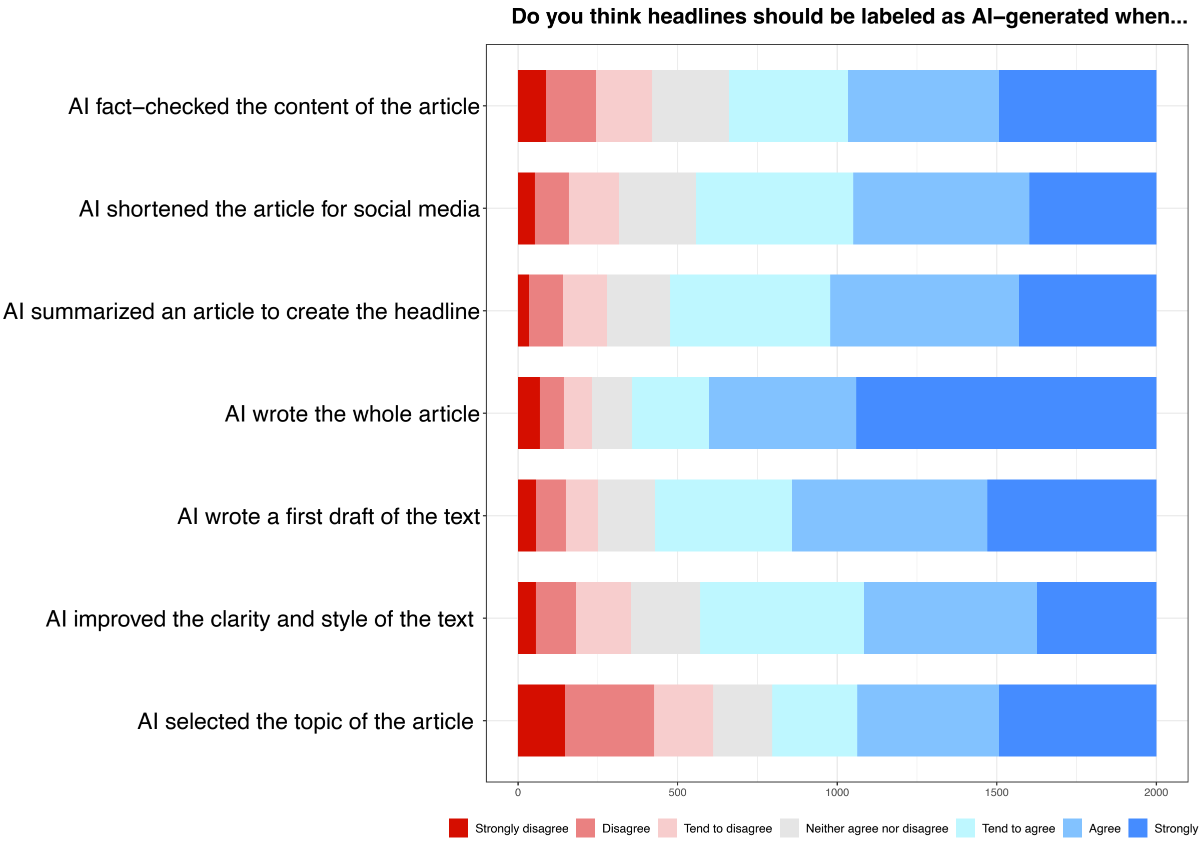

Figure S12. What kinds of AI-uses participants think should be labeled? (RQ3).

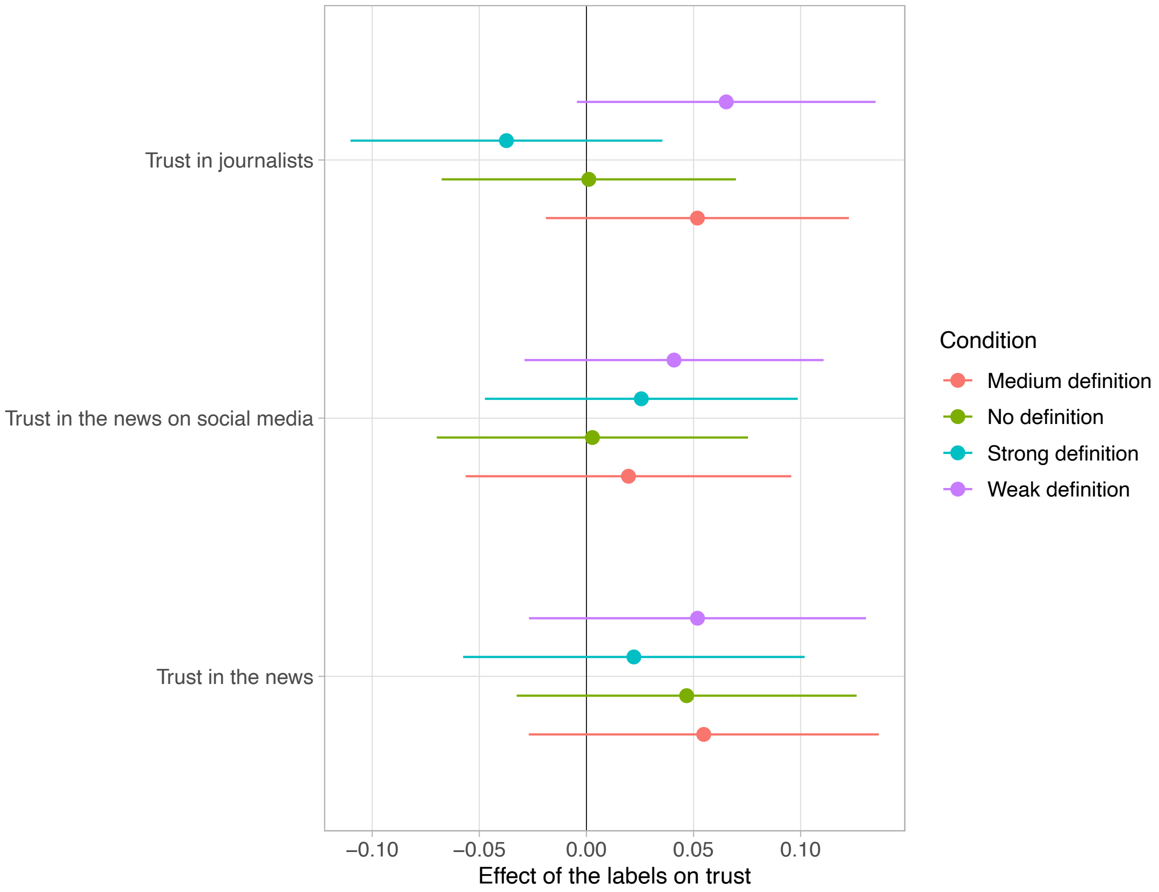

Figure S13. Effect of being exposed to headlines labeled as AI-generated on various forms of trust in the news (while controlling for trust in the news before the treatment, and country).

Comparisons of what AI means across conditions (exploratory)

Here we compare what participants think it means for a headline to be AI-generated across conditions.

First, when comparing the Control Condition (no label and no definition of AI) to the No Definition Condition (in which participants saw AI-labels but were not given definitions of AI), we find no statistically significant differences. This suggest that seeing headlines labeled as AI-generated had negligeable effects on participants' understanding of what AI-generated headlines are.

Second, to isolate the effect of providing participants with definitions of what AI-generated headlines are (as opposed to seeing headlines labeled as AI-generated), we compared the No Definition Condition to conditions in which participants were given definitions of what AI-generated headlines are.

- Participants in the Strong Condition were **more** likely to think it means selecting the topic of the article ($b = 1.93$, $p < .001$), writing the whole text ($b = 0.96$, $p < .001$), and fact-checking the article ($b = 0.41$, $p < .001$).

In the Strong Condition participants were told that AI-generated means selecting the topic and writing the whole article.

- Participants in the Medium Condition were **less** likely to think it means selecting the topic of the article ($b = - 0.73$, $p < .001$), writing the whole text ($b = - 1.12$, $p < .001$), summarizing the article ($b = - 0.31$, $p < .001$), and **more** likely to think it means writing a first draft of the article ($b = 1.07$, $p < .001$).

In the Medium Condition participants were told that AI-generated means that a journalist selected the topic of the article, and that AI wrote a first draft.

- Participants in the Weak Condition were **less** likely to think it means selecting the topic of the article ($b = - 0.67$, $p < .001$), writing a first draft of the article ($b = -1.18$, $p < .001$), writing the whole text ($b = - 1.62$, $p < .001$), summarizing the article ($b = - 0.17$, $p < .001$), and **more** likely to think it, improving the clarity and style of the article ($b = 1.25$, $p < .001$).

In the Weak Condition participants were told that AI was only used to improve the clarity of the text and adapt it to the style of the news outlet, and that a journalist selected the topic of the article and wrote it.

Overall participants responded in accordance with the definitions they were given. In the Strong Condition participants reported much stronger definitions of AI than in the Weak and Medium conditions. We mostly see these results as a manipulation check. They also suggest that the distance between the Strong and the Medium conditions is much larger than the distance between the Medium and the Weak conditions.

Comparisons of what forms of AI uses should be labeled across conditions (exploratory)

Here we compare the kinds of AI uses that participants think deserve to be labeled across conditions.

First, when comparing the Control Condition (no label and no definition of AI) to the No Definition Condition (in which participants saw AI-labels but were given no definition of AI), we find no statistically significant differences. This suggest that seeing headlines labeled as AI-generated had negligeable effects on labeling perceptions.

Second, to isolate the effect of providing participants with definitions of what AI-generated headlines are (as opposed to seeing headlines labeled as AI-generated), we compared the No Definition Condition to conditions in which participants were given definitions of what AI-generated headlines are.

- Participants in the Strong Condition were **more** likely to think that headlines should be labeled when AI selected the topic (b = 1.13, p < .001), wrote the whole text (b = 0.58, p < .001), wrote a first draft (b = 0.19, p = .030), summarized the article (b = 0.18, p = .040), and fact-checked the article (b = 0.21, p = .35).

- Participants in the Medium Condition were **more** likely to think that headlines should be labeled when AI wrote a first draft (b = 0.51, p < .001), and **less** likely to think that headlines should be labeled when AI wrote the whole text (b = -0.37, p < .001).

- Participants in the Weak Condition were **more** likely to think that headlines should be labeled when AI was used to improve the style and clarity of the article (b = 0.51, p < .001), and **less** likely to think that headlines should be labeled when AI wrote the first draft of the text (b = -0.18, p = .041), wrote the whole text (b = -0.34, p < .001).

To some extent, participants responded in accordance with the definitions they were given. Such that participants were more likely to report that labels should be applied to uses that fall within the definitions they were given. For instance, compared to the No Definition Condition, participants in the Weak Condition were more likely to consider that AI is used to improve text and clarity, and were more likely to consider that such use deserve a label. What’s troubling is that they were also less likely to consider that strong AI uses, such as writing a full article, should be labeled compared to the No Definition Condition.
